# Regulating the electrocatalytic active centers for accelerated proton transfer towards efficient CO_2_ reduction

**DOI:** 10.1093/nsr/nwaf010

**Published:** 2025-01-10

**Authors:** Yunxiang Lin, Shaocong Wang, Hengjie Liu, Xue Liu, Li Yang, Xiaozhi Su, Lei Shan, Xiyu Li, Li Song

**Affiliations:** Institutes of Physical Science and Information Technology, Leibniz International Joint Research Center of Materials Sciences, Information Materials and Intelligent Sensing Laboratory of Anhui Province, Center of Free Electron Laser & High Magnetic Field, Anhui University, Hefei 230601, China; Institutes of Physical Science and Information Technology, Leibniz International Joint Research Center of Materials Sciences, Information Materials and Intelligent Sensing Laboratory of Anhui Province, Center of Free Electron Laser & High Magnetic Field, Anhui University, Hefei 230601, China; National Synchrotron Radiation Laboratory, University of Science and Technology of China, Hefei 230029, China; Institutes of Physical Science and Information Technology, Leibniz International Joint Research Center of Materials Sciences, Information Materials and Intelligent Sensing Laboratory of Anhui Province, Center of Free Electron Laser & High Magnetic Field, Anhui University, Hefei 230601, China; Institutes of Physical Science and Information Technology, Leibniz International Joint Research Center of Materials Sciences, Information Materials and Intelligent Sensing Laboratory of Anhui Province, Center of Free Electron Laser & High Magnetic Field, Anhui University, Hefei 230601, China; Shanghai Synchrotron Radiation Facility, Shanghai Advanced Research Institute, Chinese Academy of Sciences, Shanghai 201204, China; Institutes of Physical Science and Information Technology, Leibniz International Joint Research Center of Materials Sciences, Information Materials and Intelligent Sensing Laboratory of Anhui Province, Center of Free Electron Laser & High Magnetic Field, Anhui University, Hefei 230601, China; Songshan Lake Materials Laboratory, Dongguan 523808, China; School of Physical Sciences, Great Bay University, Dongguan 523000, China; National Synchrotron Radiation Laboratory, University of Science and Technology of China, Hefei 230029, China; Zhejiang Institute of Photonelectronics, Jinhua 321004, China

**Keywords:** α-MoC_1−x_, CO_2_ reduction reaction, Joule heating synthesis, proton-coupled electron transfer process, hydrogen-bonding interaction

## Abstract

The electrochemical CO_2_ reduction reaction (CO_2_RR) is an important application that can considerably mitigate environmental and energy crises. However, the slow proton-coupled electron transfer process continues to limit overall catalytic performance. Fine-tuning the reaction microenvironment by accurately constructing the local structure of catalysts provides a novel approach to enhancing reaction kinetics. Here, cubic-phase α-MoC_1−x_ nanoparticles were incorporated into a carbon matrix and coupled with cobalt phthalocyanine molecules (α-MoC_1−x_–CoPc@C) for the co-reduction of CO_2_ and H_2_O, achieving an impressive Faradaic efficiency for CO close to 100%. Through a combination of *in-situ* spectroscopies, electrochemical measurements, and theoretical simulations, it is demonstrated that α-MoC_1−x_ nanoparticles and CoPc molecules with optimized local configuration serve as the active centers for H_2_O activation and CO_2_ reduction, respectively. The interfacial water molecules were rearranged, forming a dense hydrogen bond network on the catalyst surface. This optimized microenvironment at the electrode–electrolyte interface synergistically enhanced water dissociation, accelerated proton transfer, and improved the overall performance of CO_2_RR.

## Teaser

Incorporating adjacent cubic phase molybdenum carbide nanoparticles can regulate the interface structure among CoPc molecules and promote the proton transfer during electrocatalytic CO_2_ reduction.

## INTRODUCTION

The electrocatalytic CO_2_ reduction reaction (CO_2_RR) is widely recognized as one of the most promising approaches to mitigate the growing environmental and energy challenges owing to its technical and economic viability [1–6]. In terms of reaction pathways, energy efficiency, and technological maturity, the conversion of CO_2_–CO is considered a competitive option for industrial applications [7–10]. Advancing electrocatalysts with high selectivity and activity represents a cutting-edge area of research in CO_2_RR. The primary challenge lies in the careful design of the catalyst's structure, as well as optimizing the reaction pathways and the microenvironment of the electrode. While noble metal-based catalysts (such as Au and Ag) have shown excellent CO selectivity and low overpotential [11,12], their high cost and limited availability still hinder their large-scale use. As promising candidates, 3d transition metal-based single-atom catalysts featuring traditional metal-N_4_ catalytic centers and low costs have been extensively researched [13–15]. Furthermore, various strategies have been proposed to enhance CO_2_RR performance by lowering the energy barrier of the rate-determining step. These strategies include constructing asymmetric coordination configurations [16], introducing heterometal centers [17], and optimizing the electrode–electrolyte interface [18]. However, achieving highly efficient and selective CO_2_RR to CO remains a considerable challenge owing to the interdependence of the adsorption energies of ^*^CO_2_ and ^*^CO, which affects the formation of ^*^COOH and the desorption of ^*^CO [19,20].

In fact, the CO_2_RR is a proton-coupled electron transfer (PCET) process, where protons originating from water molecules at the electrolyte–electrode interface play a crucial role in the reaction [21–23]:


\begin{eqnarray*}
&&{\mathrm{C}}{{\mathrm{O}}_2} \,+\, {^*}\, +\, {{\mathrm{e}}^ - }{\, \to\, ^*}{\mathrm{C}}{{\mathrm{O}}_2}^ - ,\\
&& ^*{\mathrm{C}}{{\mathrm{O}}_2}^ - \, +\, {{\mathrm{H}}^*}{\,\, \to\, ^*}{\mathrm{COOH}},\\
&& ^*{\mathrm{COOH}} \,+\, {{\mathrm{e}}^ - } \,+\, {{\mathrm{H}}^*}{\, \to\, ^*}{\mathrm{CO}} + {{\mathrm{H}}_2}{\mathrm{O}},\\
&& ^*{\mathrm{CO}} \to {\mathrm{CO}}{\, +\, ^*}.
\end{eqnarray*}

Hence, in addition to reducing energy barriers, enhancing the kinetics of the PCET steps is an important strategy to enhance CO_2_RR [24,25]. One method involves introducing long-chain molecules into the electrolyte to modify the hydrogen bond network at the electrode surface during the CO_2_RR process. For instance, Li and colleagues showed that quaternary ammonium salt surfactants arrange themselves systematically when an external voltage is applied during CO_2_RR, creating a locally hydrophobic environment. This leads to an optimized electric double layer, ultimately enhancing both activity and selectivity [18]. Another approach involves creating neighboring active sites (e.g. adjacent single-atom sites) to enhance water dissociation and ensure an adequate supply of protons [19]. This strategy of regulating dissociating water molecules to improve catalytic performance can considerably expand the potential for material design and fabrication in complex PCET processes.

Here, inspired by the strategy of enhancing the PCET process through optimized water dissociation, α-MoC_1−x_ nanoparticles, known for their excellent water adsorption and dissociation capacity, were selected to modify the adjacent microenvironment of cobalt phthalocyanine (denoted as α-MoC_1−x_–CoPc@C) to facilitate improved CO_2_ to CO conversion. The resulting α-MoC_1−x_–CoPc@C demonstrates a high Faradaic efficiency for CO (FE_CO_) and impressive stability. A series of *in-situ* characterizations further confirmed the rearrangement of interface water and the rapid transformation of intermediates. Additionally, theoretical simulations indicate that the incorporation of α-MoC_1−x_ nanoparticles can effectively change the adsorption behavior of water molecules near the CoPc molecules, resulting in accelerated proton transfer in the hydrogen bond network. This work presents a promising approach to interface water rearrangement through the rational design of catalyst structures aimed at accelerating the PCET process.

## RESULTS AND DISCUSSION

### Structure characterization

**Figure 1.**
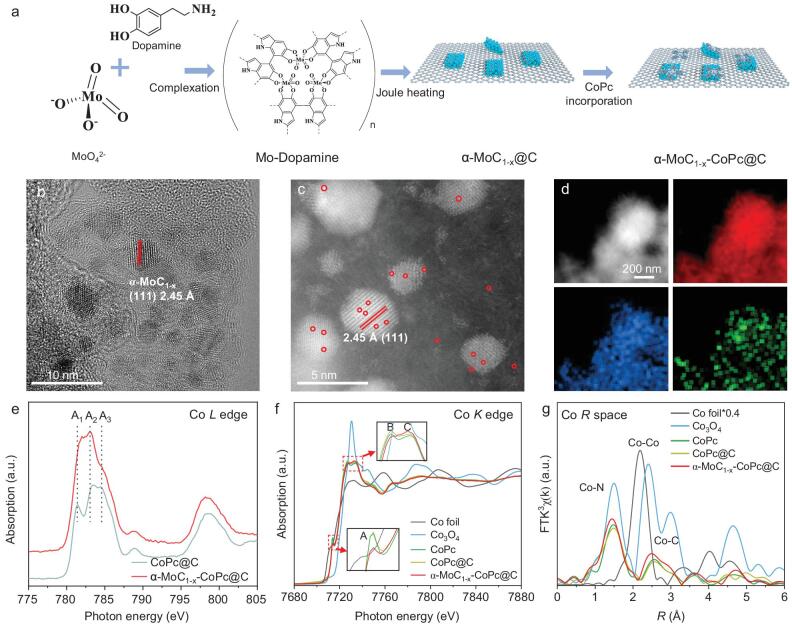
Synthesis and characterizations for as-prepared samples. (a) Schematic representation of the synthesis of α-MoC_1−x_–CoPc@C. (b) HRTEM and (c) HAADF–STEM images of α-MoC_1−x_–CoPc@C. (d) Elemental mapping images of α-MoC_1−x_–CoPc@C, where red, blue and green indicate C, Mo and Co, respectively. (e) Co *L*-edge XANES spectra of α-MoC_1−x_–CoPc@C and its counterparts. (f) Co *K*-edge XANES spectra. (g) FT–EXAFS curves of Co *K*-edge presented in R space for α-MoC_1−x_–CoPc@C and its counterparts.

The α-MoC_1−x_–CoPc@C catalyst was synthesized through the following steps (Fig. 1a) [26]: (1) reacting ammonium molybdate with dopamine to create the Mo–dopamine precursor, (2) obtaining the graphene-supported α-MoC_1−x_ nanoparticles (α-MoC_1−x_@C) via rapid Joule heating at 1500°C for 20 s, and (3) anchoring the CoPc molecules onto the α-MoC_1−x_@C substrate. Previous studies have shown that atomically dispersed metal phthalocyanine on a carbon-based substrate can induce dipole forces owing to π–π interactions, thereby changing the electron density of the metal centers [27,28]. The α-MoC_1−x_@C substrate shows an urchin-like morphology formed by a carbon framework supporting α-MoC_1−x_ nanoparticles (Fig. S1). Notably, the introduction of CoPc into the α-MoC_1−x_@C substrate has a minimal impact on the morphology of the α-MoC_1−x_–CoPc@C (Fig. S2). Additionally, the α-MoC_1−x_ nanoparticles have an average size of ∼5 nm and exhibit a lattice fringe of 2.45 Å, corresponding to the (111) plane (Fig. 1b) [29]. High-angle annular dark-field scanning transmission electron microscopy (HAADF–STEM) images of the prepared samples were obtained to examine the morphological properties. Owing to Mo's higher atomic number (Z) compared to Co, the Mo atoms appear as brighter spots in the HAADF–STEM image, which makes it challenging to resolve the Co atoms. Therefore, we adjusted the tilt angle and defocus to differentiate the contrast between Co and Mo atoms. As shown in Fig. 1c, the HAADF–STEM image of α-MoC_1−x_–CoPc@C reveals bright dots on the surfaces of both the α-MoC_1−x_ nanoparticles and the carbon matrix. This observation suggests that CoPc molecules are likely located on both the carbon matrix and the surface of α-MoC_1−x_. These findings indicate the atomic dispersion of CoPc across the α-MoC_1−x_@C substrate. Furthermore, to better understand the structural characteristics between CoPc and the α-MoC_1−x_@C substrate, electron energy loss spectroscopy was performed. As depicted in Fig. S3, signals corresponding to both Co and Mo *L*-edges were detected in the selected area, confirming the successful atomic dispersion of CoPc on the α-MoC_1−x_@C substrate. For comparison, carbon-supported CoPc (CoPc@C) was prepared without the addition of α-MoC_1−x_ nanoparticles, revealing similarly single-dispersed Co sites, as shown in Fig. S4. Additionally, the elemental mapping images further confirmed the uniform distribution of C, Mo and Co atoms in α-MoC_1−x_–CoPc@C (Fig. 1d). The α-MoC_1−x_@C substrate and CoPc@C counterparts exhibited similar elemental dispersion, suggesting a consistent synthetic strategy (Figs S5 and S6). The X-ray diffraction patterns also indicated successful CoPc loading without structural damage during annealing, while the incorporated α-MoC_1−x_ nanoparticles displayed a cubic phase structure (Fig. S7). The mass loading of Co atoms in α-MoC_1−x_–CoPc@C and CoPc@C, measured by inductively coupled plasma mass spectrometry (ICP–MS), is 0.99 and 1.11 wt%, respectively, indicating a consistent metal loading of active centers for CO_2_RR (Table S1). X-ray photoelectron spectroscopy (XPS) was used to analyze the surface valence states. The C and N 1s spectra of the various samples demonstrate the successful integration of CoPc into the α-MoC_1−x_@C substrate, with distinct peaks of CoPc visible in α-MoC_1−x_–CoPc@C (Figs S8 and S9). After loading CoPc, there was a decrease in the Mo^2+^ content, while the levels of Mo^4+^ and Mo^6+^ increased, suggesting a charge transfer between the substrate and CoPc (Fig. S10). Additionally, the Co^3+^ XPS peak diminished after incorporation into the α-MoC_1−x_@C substrate, which aligns with the observed trend of increasing average valence state of Mo (Fig. S11).

Considering the direct relationship between the spin state of Co and its catalytic performance, we measured the Co *L*-edge X-ray absorption near-edge structure (XANES). As shown in Fig. 1e, three peaks labeled A_1_ to A_3_ are visible in the *L_3_*-edge region of the Co *L*-edge XANES spectra. The A_1_ peak is primarily attributed to the transitions from the 2*p*_3/2_ to the 3*d_z^2^_* orbitals, while the A_2_ and A_3_ peaks stem from transitions between the 2*p*_3/2_ and 3*d_x^2^_–_y^2^_* orbitals [30]. Notably, α-MoC_1−x_–CoPc@C exhibits a higher intensity for the A_1_ peak and a lower intensity for the A_3_ peak compared to CoPc@C. The increased intensity of the A_1_ peak may indicate a more favorable interaction between the vertical 3*d_z^2^_* orbitals and the substrate, while the reduced intensity of the A_3_ peak could be attributed to enhanced charge transfer facilitated by the neighboring α-MoC_1−x_ nanoparticles. Hence, the rearranged *L*-edge XANES spectrum of Co atoms indicated the strong π–π interaction between substrate and CoPc molecules. The Co *K*-edge XANES spectra of α-MoC_1−x_–CoPc@C and CoPc@C show similar patterns, with some differences in the intensity of characteristic peaks (Fig. 1f). This similarity suggests that both have comparable *D*_4*h*_ asymmetric geometric structures and valence states. Notably, the pre-edge peak A (1*s* to 4*p_z_* transition), which is indicative of a square–planar Co–N_4_ structure, is lower in α-MoC_1−x_–CoPc@C compared to CoPc and CoPc@C. This reduction implies a distortion in the *D*_4*h*_ symmetry of the Co–N_4_ structure. Additionally, the intensity of peak C, which arises from the 1*s* to 4*p_x,y_* transition and multiple scattering, has increased upon incorporation with the α-MoC_1−x_@C substrate. This enhancement may positively impact the subsequent reaction processes [31]. The C *K*-edge and N *K*-edge XANES spectra of the as-prepared samples further confirmed the local electron rearrangement (Fig. S12) [32,33]. Additionally, the Fourier transformed extended X-ray absorption fine structure (FT–EXAFS) curves were analyzed to investigate the local configuration of Co. Because integrating CoPc with the α-MoC_1−x_@C substrate may cause CoPc to disperse across the carbon matrix and the α-MoC_1−x_ surface, understanding the local structure of Co atoms is crucial. As shown in Fig. 1g, the α-MoC_1−x_–CoPc@C shows a slightly shorter bond length for the first shell Co–N bond and the second shell Co–C bond compared to CoPc and CoPc@C [34,35]. This variation may result from molecular distortion owing to electrostatic adsorption. In particular, the comparison of FT–EXAFS curves for α-MoC_1−x_–CoPc@C and CoPc revealed that the local structure of CoPc molecules is considerably influenced by the incorporation of α-MoC_1−x_ nanoparticles into the carbon matrix. The EXAFS fitting results confirmed the coordination environment of Co for α-MoC_1−x_–CoPc@C (Fig. S13, Table S2). Based on our discussions about the morphology and electronic structure of the as-prepared samples, we can conclude that the incorporation of CoPc onto the α-MoC_1−x_@C substrate has substantially changed the local configuration of CoPc. The presence of α-MoC_1−x_ nanoparticles in the catalyst has improved the π–π interactions between CoPc and the α-MoC_1−x_@C substrate, leading to enhanced interactions that may further enhance the catalytic performance of the active centers.

### Electrochemical CO_2_RR performance

**Figure 2.**
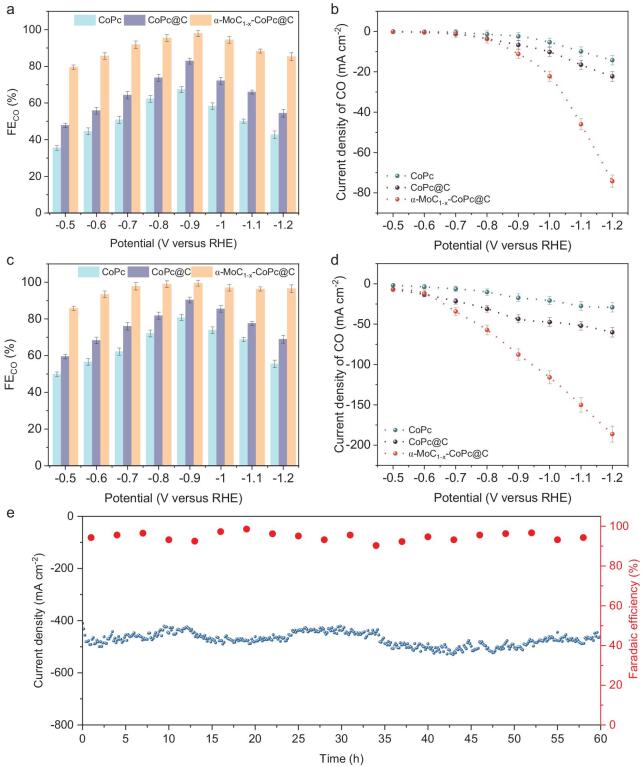
Evaluation of the electrocatalytic CO_2_RR performance. (a) The FE_CO_ at various operational potentials for α-MoC_1−x_–CoPc@C, CoPc@C, and CoPc assessed in H-cell measurements using CO_2_-saturated 0.5 M KHCO_3_ as the electrolyte. (b) The CO current density evaluated alongside the FE and reaction current densities at different potentials in the H-cell. (c) The FE_CO_ at different operational potentials for α-MoC_1−x_–CoPc, CoPc@C, and CoPc in flow-cell measurements with 1 M KOH as the electrolyte. (d) Corresponding current densities of CO in flow-cell. (e) Stability test for α-MoC_1−x_–CoPc performed at a current density of 500 mA cm^−2^ under flow-cell testing conditions.

The electrocatalytic performance of various as-prepared samples for CO_2_RR was first evaluated in a CO_2_-saturated 0.5 M KHCO_3_ electrolyte using a homemade H-type electrochemical cell. The gas products were CO and H_2_, with no liquid products detected in α-MoC_1−x_–CoPc@C and CoPc@C, while only H_2_ was detected in α-MoC_1−x_@C and the C substrate. Therefore, the CO_2_RR performance of CoPc, CoPc@C, and α-MoC_1−x_–CoPc@C was examined in the subsequent discussions on catalytic performance. As shown in Fig. S14, all the three samples exhibited a higher current density when CO_2_ was introduced into the electrolyte compared to when Ar was used, indicating their capacity for CO_2_ reduction. It is important to highlight that the maximum current density of α-MoC_1−x_–CoPc@C reaches 87.5 mA cm^−2^, which is considerably greater than those of CoPc (42.9 mA cm^−2^) and CoPc@C (32.6 mA cm^−2^) at a potential of −1.2 V_RHE_. The total FE_CO_ values and H_2_ across all catalysts are nearly 100% at the tested potentials. Furthermore, the FE_CO_ was calculated to compare the CO_2_RR performance of the prepared samples, as shown in Fig. 2a. The α-MoC_1−x_–CoPc@C demonstrates a high FE_CO_ exceeding 90% in a potential range of −0.7 to −1.0 V_RHE_, achieving a peak FE_CO_ of 97.9% at −0.9 V_RHE_, which is considerably higher than that of CoPc and CoPc@C. In addition to selectivity, the partial current density for CO (*j*_CO_) was calculated to evaluate the CO_2_RR performance of the obtained samples (Fig. 2b). The α-MoC_1−x_–CoPc@C exhibited a considerably higher *j*_CO_ compared to CoPc and CoPc@C. For instance, at −1.1 V_RHE_, the *j*_CO_ of α-MoC_1−x_–CoPc@C reached 45.98 mA cm^−2^, which is 2.79 times that of CoPc@C and 4.64 times that of CoPc. Although the *j*_CO_ is substantial at the more negative potential of −1.2 V_RHE_, the decrease in FE_CO_ may be attributed to limited CO_2_ supply and competition from the hydrogen evolution reaction (HER) [36]. Regarding the CO_2_RR performance of α-MoC_1−x_@C, it is evident that hydrogen is the primary product, while CO can be practically disregarded. This suggests that α-MoC_1−x_@C exhibits inert behavior in CO_2_RR (Fig. S16). We also assessed the CO_2_RR performance of α-MoC_1−x_–CoPc@C in a flow cell by spurting the catalysts to a gas diffusion electrode (GDE), which enhances gas diffusion and mass transfer considerably [37–39]. Notably, the GDE-supported catalysts demonstrated a high FE_CO_ exceeding 90% across a broad potential range from −0.6 to −1.2 V_RHE_, reflecting the excellent selectivity of α-MoC_1−x_–CoPc@C (Fig. 2c and Fig. S15). Additionally, the *j*_CO_ of α-MoC_1−x_–CoPc@C was considerably increased to nearly 200 mA cm^−2^, while maintaining a high FE_CO_ >90% (Fig. 2d). To assess the catalyst's stability, we performed chronoamperometry tests at a high current density of ∼500 mA cm^−2^. As shown in Fig. 2e, α-MoC_1−x_–CoPc@C demonstrates impressive stability in current density and maintains a high FE_CO_ over a 60-h test period, indicating excellent durability. Therefore, the evaluation of CO_2_RR performance further suggests that the α-MoC_1−x_ nanoparticles in α-MoC_1−x_–CoPc@C may enhance reaction kinetics related to proton transfer and intermediate transfer rather than acting solely as catalytic centers.

### Mechanism investigation using *in-situ* characterization

**Figure 3.**
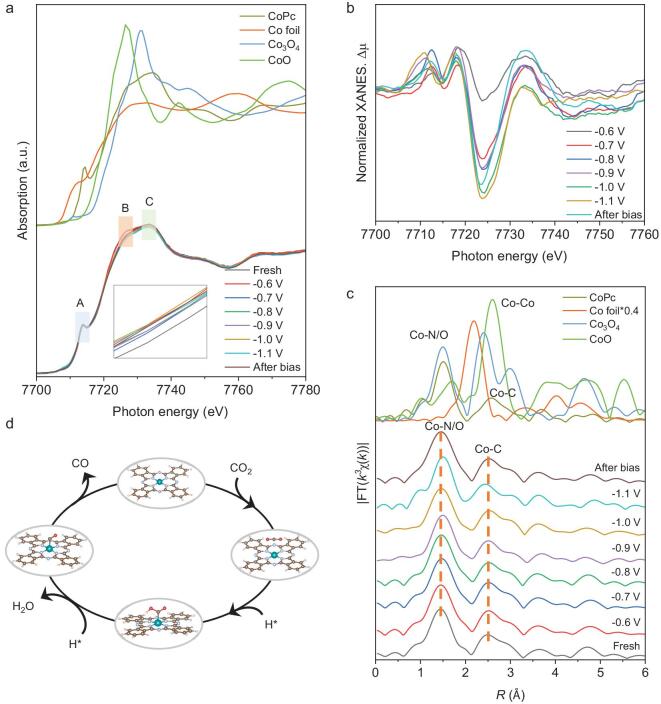
*In-situ* XAFS measurements of Co *K*-edge. (a) Co *K*-edge XANES spectra of α-MoC_1−x_–CoPc@C at various operational potentials, along with the corresponding standard references. (b) Normalized differential XANES spectra for the Co *K*-edge. (c) k^3^-weighted FT–EXAFS curves at different potentials. (d) Illustration of the CO_2_RR pathway at the Co sites.

*In-situ* XAFS measurements were performed to further explore the origins of the impressive catalytic performance of α-MoC_1−x_–CoPc@C during the CO_2_RR process [40,41]. As shown in Fig. 3a, the changed I_B_/I_C_ value indicates enhanced catalytic activity. Notably, the absorption edge at −0.6 V_RHE_ shifts to lower photon energy compared to the fresh sample, which results from the strong interaction between the adsorbed CO_2_ molecules and Co atoms. When the potential is applied between −0.7 and −0.9 V_RHE_, the absorption edges experience a slight increase before stabilizing, suggesting electron transfer from Co to ^*^COOH and the generation of CO. Additionally, when the voltage was set to −1.0 V_RHE_, the absorption edge continued to shift toward lower energy. This shift may result from the competitive occurrence of the HER reaction and the accumulation of electrons at the cathode, which keeps the Co atoms in a reduced oxidation state. When the applied potential is reversed, the absorption edge slightly shifts to higher photon energy, possibly owing to the desorption of intermediates and the release of accumulated electrons [42]. These variations in the Co *K*-edge are more clearly illustrated in the difference curves shown in Fig. 3b, where a consistent pattern in accordance with the absorption edge can be observed. The FT–EXAFS curves were further analyzed to reveal the local geometric configuration. As shown in Fig. 3c, the scattering peak at ∼1.5 Å for Co is attributed to the first shell Co–N/O bond, while the scattering peak at 2.5 Å originates from the second shell Co–C bond. Notably, the bond length of the Co–N/O bond slightly increased by ∼0.02 Å when transitioning from −0.7 to −0.9 V_RHE_, which aligns with the robust CO_2_RR process occurring at the working potential. Additionally, the EXAFS fitting results further confirmed that the coordination number of Co–N/O exceeds four during the CO_2_RR process, indicating the adsorption of CO_2_ molecules and intermediates (Fig. S17, Table S2). The coordination number and bond length of the second shell Co–C bond also changed under the working potentials. Given the planar molecular structure of CoPc, these changes in the Co–C bond may be attributed to out-of-plane vibrations. Thus, we can conclude that Co atoms act as the catalytic centers during the CO_2_RR process, accompanied by the dynamic transformation of intermediates (Fig. 3d).

Considering that the CO_2_RR is a PCET process, water activation and protonation are crucial factors that can substantially impact catalytic performance and selectivity toward CO production [43,44]. *In-situ* synchrotron radiation-based attenuated total reflectance surface-enhanced infrared absorption spectroscopy (ATR–SEIRAS) was performed to examine the dynamic evolution of interfacial water and other intermediates. The series of peaks observed at ∼1650, 1450 and 1370 cm^−1^ are attributed to H_2_O, ^*^COOH and ^*^HCO_3_, respectively [45]. The infrared peak ∼2100 cm^−1^ is likely associated with the generated CO bonded to the catalyst's surface via top-site adsorption, which may facilitate the subsequent desorption of the CO product on flat CoPc molecules [28,46]. Additionally, we observe a red shift of the ^*^CO band at more negative potentials on α-MoC_1−x_–CoPc@C during CO_2_RR. This shift could be attributed to the Stark tuning effect, which arises from potential-induced energy level modulation and enhanced charge transfer at higher reduction potentials (Fig. S18a) [47–50]. Furthermore, the intensity of the ^*^CO peak on CoPc@C is greater than that on α-MoC_1−x_–CoPc@C, indicating a higher coverage of ^*^CO species on CoPc@C and a slower product desorption compared to α-MoC_1−x_–CoPc@C (Fig. S18b) [51]. Therefore, it can be concluded that α-MoC_1−x_–CoPc@C demonstrates a faster charge transfer and product desorption capacity than CoPc@C, which further supports the accelerated reaction kinetics resulting from the incorporation of α-MoC_1−x_.

**Figure 4.**
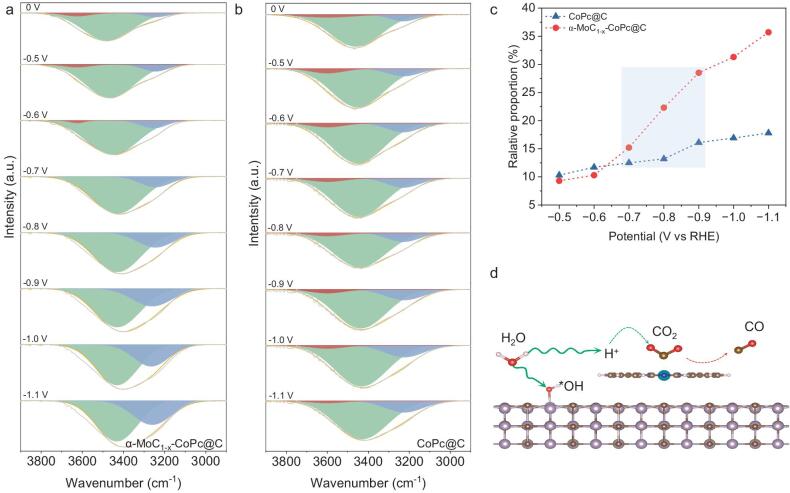
Investigation of the interfacial water by ATR–SEIRAS. *In-situ* ATR–SEIRAS spectra showing the interfacial water structure on (a) α-MoC_1−x_–CoPc and (b) CoPc@C during CO_2_RR. (c) The potential-dependent relative proportion of interfacial 4-HB·H_2_O for α-MoC_1−x_–CoPc and CoPc@C (note: the relative proportion of 4-HB·H_2_O was determined by dividing the proportion of each component obtained from Gaussian fitting). (d) A schematic diagram illustrating the CO_2_RR reaction process over α-MoC_1−x_–CoPc@C.

The dynamic structure of interfacial water can be examined through the O–H stretching band observed in the ATR–SEIRAS spectra, which ranges from 3000 to 3600 cm^−1^ [19,52,53]. This O–H stretching band can be deconstructed into three components using Gaussian fitting, with bands at 3250, 3450 and 3650 cm^−1^ corresponding to 4-coordinated, 2-coordinated hydrogen-bonded, and K^+^ hydrated free water, respectively (denoted as 4-HB·H_2_O, 2-HB·H_2_O and K^+^·H_2_O) [19,52,53]. The 4-HB·H_2_O is often referred to as ice water owing to its dense hydrogen bond network, which may directly affect the electric double layer structure and hinder hydrogen coupling to H_2_ [54,55]. As shown in Fig. 4a and b, varying amounts of these types of water can be observed at different working potentials, indicating a rearrangement of the interfacial water structure during the CO_2_RR process. The content of 4-HB·H_2_O considerably increases when the operating potential reaches −0.7 V_RHE_ for α-MoC_1−x_–CoPc@C, which can be attributed to the formation of a dense hydrogen bond network at the electrode–electrolyte interface [19]. In contrast, the amount of K^+^·H_2_O for α-MoC_1−x_–CoPc@C becomes negligible when the applied potential exceeds −0.7 V_RHE_, while K^+^·H_2_O is detected throughout the entire potential range for CoPc@C. This difference may result from the enhanced adsorption of water molecules and the creation of a denser hydrogen bond network at the electrode–electrolyte interface induced by α-MoC_1−x_ nanoparticles. Additionally, the relative proportions of 4-HB·H_2_O were calculated to explore intrinsic correlations (Fig. 4c). The content of 4-HB·H_2_O at the electrode–electrolyte interface of α-MoC_1−x_–CoPc@C is nearly double that of CoPc@C when the potentials are below −0.8 V_RHE_. This indicates that α-MoC_1−x_ can effectively rearrange the adsorption behavior of water molecules and the hydrogen bond network as the applied potentials increase. Because ATR–SEIRAS measurements are sensitive to the surface intermediates on the electrocatalysts, the lack of a K^+^·H_2_O signal on α-MoC_1−x_–CoPc@C at potentials lower than −0.7 V_RHE_ may be attributed to the dominance of 4-HB·H_2_O, which limits the presence of K^+^·H_2_O.

To gain a comprehensive understanding of the crucial role of water activation in the CO_2_RR process, kinetic isotope effect (KIE) measurements using H_2_O and D_2_O as proton sources were performed on the prepared samples [56]. Figure S19 shows that the KIE values for α-MoC_1−x_–CoPc@C and CoPc@C are both ∼2, suggesting that the slow activation of H_2_O impedes the reaction process [20]. Furthermore, α-MoC_1−x_–CoPc@C exhibits a lower KIE value compared to CoPc@C, which can be attributed to an enhanced dissociation of H_2_O on α-MoC_1−x_–CoPc@C [57]. Thus, the reduced KIE values indicate that the incorporation of α-MoC_1−x_ nanoparticles promotes water dissociation, thereby accelerating proton transfer. Furthermore, we performed electrochemical impedance spectroscopy to examine the dynamic behavior at the electrode–electrolyte interface. When using the D_2_O-replaced electrolyte, a larger semi-circle and phase angle were observed (Fig. S20, Tables S3–S6), indicating a considerable charge transfer resistance (*R*_ct_) and mass transfer limitations owing to H_2_O dissociation. We also analyzed the Bode plots at different working potentials to assess mass transfer over α-MoC_1−x_–CoPc@C and CoPc@C [18]. As shown in Fig. S21a and b, the phase angle gradually shifts to higher frequencies with increasing applied potentials for α-MoC_1−x_–CoPc@C, whereas no notable changes occur for CoPc@C. This implies that proton generation from water dissociation is more favorable on α-MoC_1−x_–CoPc@C [58]. The next key aspect to address is the investigation of proton transfer mechanisms on the surfaces of catalysts. As shown in Fig. S21c and d, a considerably lower frequency and decreased amplitude of phase angle (Ø_peak_) are observed for α-MoC_1−x_–CoPc@C compared to CoPc@C, indicating its superior proton transfer capability [58]. Given the enhanced performance in CO_2_RR and a denser hydrogen bond network at operating potentials, it can be inferred from the preceding discussion that the reaction process consists of the following steps: (1) α-MoC_1−x_ activates water molecules to generate protons, (2) protons are transferred to the catalytic centers via a dense hydrogen bond network, and (3) the rapid proton transfer accelerates the transformation of intermediates and the reaction kinetics (Fig. 4d).

### Theoretical calculations

**Figure 5.**
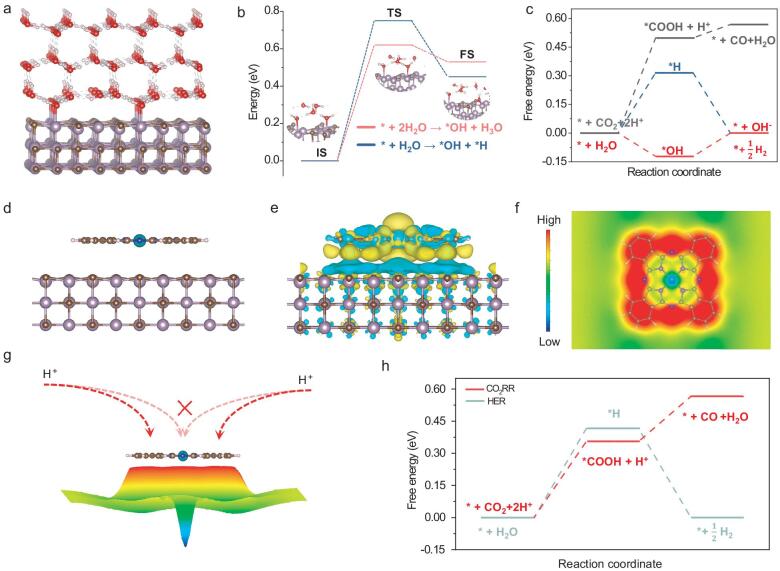
Theoretical simulations of the reaction mechanisms. (a) The atomic structure of ice-like water on the α-MoC_1−x_ surface. (b) The calculated activation energies for water splitting to produce hydrated hydrogen (*H_3_O) and adsorbed hydrogen (*H). (c) The calculated free energies for CO_2_RR, HER, and water-splitting into protons (H^+^) and hydroxyl ions (OH^−^) on the α-MoC_1−x_ surface. (d) The atomic structure of CoPc loaded on the α-MoC_1−x_ surface. (e) The top views of the isosurface charge density of CoPc on the α-MoC_1−x_ surface (isovalue: 0.001 eV/Å^3^). (f) The 2D electric potential map at a vertical height of ∼2 Å above the Co site. (g) The schematic representation of proton diffusion. (h) The calculated free energy profiles for CO_2_RR and HER at the Co site.

Density functional theory calculations were performed to explore the CO_2_RR mechanism, focusing on interface water activation and proton transfer during the reaction. Based on the experimental validation of 4-HB·H_2_O through ATR–SEIRAS measurements, the simulated structure of 4-HB·H_2_O on the α-MoC_1−x_ surface can be interpreted as an ice-like water configuration, which facilitates proton transfer rather than coupling (Fig. 5a) [59]. The activation energies for water dissociation to form hydrate protons (H_3_O) and adsorbed hydrogen (^*^H) are calculated to be 0.62 and 0.75 eV, respectively (Fig. 5b, Figs S22 and S23) [60]. Importantly, the lower activation energy for H_3_O compared to that for ^*^H suggests that the water dissociation process is more likely to produce H_3_O in the hydrogen bond network at the interface of 4-HB·H_2_O and the α-MoC_1−x_ surface rather than generating ^*^H. This is advantageous for suppressing ^*^H coupling. Furthermore, in comparison to the free energy of CO_2_RR (0.49 eV), water dissociation into protons and hydroxyl ions is expected to occur more readily on the α-MoC_1−x_ surface (Fig. 5c, Figs S24 and S25). These results indicate that α-MoC_1−x_ primarily facilitates water activation rather than CO_2_RR, which aligns with earlier evaluations of catalytic performance. To clarify the key role of CoPc in CO_2_RR, we simulated the local electronic structure of α-MoC_1−x_–CoPc@C (Fig. 5d). Notably, there is a positive correlation between the negative charge density accumulated on CoPc and the transfer rate of positively charged protons. Additionally, the negative charge on CoPc can shorten the proton diffusion path to the reactive site by inducing a specific directionality in proton transfer. Consequently, the charge transfer behavior in the α-MoC_1−x_–CoPc@C system results in a negatively charged CoPc molecule, which enhances reaction dynamics. However, establishing a quantifiable relationship between the catalyst's charge density and the proton transfer rate is essential for guiding the development of new catalysts. Molecular engineering of CoPc, such as group modifications, can be used to achieve precise adjustments of the charge density of CoPc molecules loaded on the MoC substrate, facilitating the exploration of the specific relationship between charge density and proton transfer rate. The charge density plots demonstrate strong charge transfer between α-MoC_1−x_ and the CoPc molecule, supporting the findings from the XAFS analysis (Fig. 5e). Additionally, the specific incorporation features between α-MoC_1−x_@C and CoPc may generate a strong built-in electric field that aids proton diffusion toward the CoPc molecules. In general, positively charged protons tend to accumulate in regions of high potential energy [61]. As shown in Fig. 5f, the 2D electric potential map at a vertical height of ∼2 Å above the Co site indicates that the high-potential-energy region surrounds the Co site. This suggests that protons generated on the α-MoC_1−x_ surface are likely to diffuse directionally and remain around the Co site rather than in the vertical space above it (Fig. 5g). The presence of protons around the Co site facilitates the hydrogenation steps in the CO_2_RR process. Fig. 5h shows that the free energy profiles reveal an energy barrier for CO_2_RR of 0.35 eV, which is lower than the 0.41 eV barrier for HER, indicating that CO_2_RR is more thermodynamically favorable than HER. Meanwhile, the free energy results for CO_2_RR and HER indicated that HER was inhibited, which aligns with the experimental findings. Therefore, we can summarize the strong catalytic performance of α-MoC_1−x_–CoPc@C for CO_2_RR as follows. (1) Dense hydrogen-bond network under working potentials: The increased presence of 4-HB·H_2_O, generated by the α-MoC_1−x_ nanoparticles, contributes to the formation of a dense hydrogen-bond network under working conditions, which prevents the dissociation of free water and the coupling of protons. (2) Easier formation of hydrated protons compared to adsorbed protons: The lower activation energy for H^+^ formation (simulated by H_3_O) relative to that of ^*^H indicates that protons are more likely to form hydrated protons through water splitting and remain in the hydrogen-bond network, rather than coupling to create hydrogen molecules. (3) Fast proton transfer through a dense hydrogen-bond network: The dense hydrogen-bond network at the electrolyte/electrode interface provides a rapid proton transfer pathway, which accelerates the subsequent hydrogenation of intermediates in CO_2_RR.

## CONCLUSION

In summary, we have developed α-MoC_1−x_ nanoparticles supported by a carbon matrix that integrate with the CoPc catalyst. This composite demonstrates exceptional CO_2_ reduction activity to CO, achieving a FE_CO_ close to 100%, along with excellent stability. Synchrotron radiation X-ray spectroscopies revealed that the incorporation of α-MoC_1−x_ considerably changed the local electronic structure of cobalt, leading to an optimized local configuration. A series of *in-situ* characterizations, in conjunction with theoretical simulations, show that the α-MoC_1−x_ nanoparticles can greatly enhance the adsorption and dissociation of interfacial water, forming a dense hydrogen bond network that facilitates rapid proton transfer. Our findings highlight that structuring the interfacial water through carefully designed catalyst components can considerably enhance CO_2_RR performance.

## Supplementary Material

nwaf010_Supporting_Information-revised
