# Supplementary material for: Regulating the electrocatalytic active centers for accelerated proton transfer towards efficient CO2 reduction
Source: Natl Sci Rev. 2025 Jan 10;12(3):nwaf010. doi: 10.1093/nsr/nwaf010 (PMC11879416; doi:10.1093/nsr/nwaf010)
Supplement: nwaf010_Supporting_Information-revised [file nwaf010_Supporting_Information-revised.pdf]

Regulating the cobalt phthalocyanine molecules by introducing adjacent cubic molybdenum carbide nanoparticles for accelerated proton transfer towards efficient CO_2 reduction reaction

Yunxiang Lin[1], Shaocong Wang[1], Hengjie Liu[2, *], Xue Liu[1], Li Yang[1], Xiaozhi Su[3], Lei Shan[1], Xiyu Li[4, 5, *], Li Song[2, 6, *]

[1]Institutes of Physical Science and Information Technology, Leibniz International Joint Research Center of Materials Sciences, Information Materials and Intelligent Sensing Laboratory of Anhui Province, Center of Free Electron Laser & High Magnetic Field, Anhui University, Hefei, 230601, China.

[2]National Synchrotron Radiation Laboratory, University of Science and Technology of China, Hefei, 230029, China

[3]Shanghai Synchrotron Radiation Facility, Shanghai Advanced Research Institute, Chinese Academy of Sciences, Shanghai 201204, China

[4]Songshan Lake Materials Laboratory, Dongguan, Guangdong 523808, P. R. China

[5] Great Bay university, Dongguan, Guangdong 523000, China

[6] Zhejiang Institute of Photonelectronics, Jinhua, Zhejiang 321004, China

[*]**Corresponding authors.** E-mails: liuhengjie@ustc.edu.cn; xylizy@ustc.edu.cn; song2012@ustc.edu.cn;

Experimental section

Chemicals

Ammonium molybdate tetrahydrate ($H_{24}Mo_7N_6O_{24}\cdot 4H_2O$, 99%), ammonium hydroxide solution ($NH_3\cdot H_2O$, 25~28%), dopamine hydrochloride ($C_8H_{11}NO_2\cdot HCl\cdot 4H_2O$, 98%), N,N-dimethylformamide (C_3H_7NO, >99.9%), potassium bicarbonate ($KHCO_3$, 99.9%), and potassium hydroxide (KOH, 95%) were purchased from Macklin Biochemical Technology Co., Ltd. (Shanghai, China). Cobalt phthalocyanine ($C_{32}H_{16}CoN_8$, 95%) was purchased from Aladdin Reagent Co., Ltd. Ethanol absolute (C_2H_6O, 99.7%) was purchased from Sinopharm Chemical Reagent Co. Nafion PFSA Polymer was purchased from Suzhou Sinero Technology Co., Ltd. Ultrapure water (resistivity of 18.2 $M\Omega\cdot cm^{-1}$) was used throughout the entire experimental procedure.

Synthesis of α-MoC_{1-x}@C:

The synthesis of the precursor of α-MoC_{1-x} is based on our previous reports with some modifications. First, 40 mg of $H_{24}Mo_7N_6O_{24}\cdot 4H_2O$ was dissolved in a mixture of 30 mL DI water and 20 mL anhydrous ethanol at room temperature with magnetic stirring for 30 minutes. Next, 0.65 mL of 25~28 wt% ammonium hydroxide was slowly added dropwise to the mixture until the pH reached 8.5~9, followed by continued magnetic stirring for 30 minutes. Then, 300 mg of dopamine hydrochloride was added, and the mixture was stirred magnetically for 12 hours to ensure complete polymerization. After the reaction, the mixture was washed repeatedly with DI water and anhydrous ethanol, centrifuged, and finally dried overnight at 60 ℃ in a vacuum oven to obtain the precursor powder. To prepare α-MoC_{1-x}, the precursor was treated by fast Joule-heating synthesis at the temperature of 1500 ℃ (100A) for 20 seconds at the Joule Heating Device (CIS-JH3.3-P, manufactured by Hefei In-situ Technology Co., LTD) under a N_2 atmosphere.

Synthesis of carbon substrate:

The synthesis of carbon substrate is essentially same as that of α-MoC_{1-x} without adding Mo salts to the mixed solution.

Synthesis of α-MoC_{1-x}-CoPc@C and CoPc@C:

First, 50 mg of the obtained α-MoC_{1-x}@C and 5 mg of CoPc are uniformly dispersed in 15 mL of DMF, respectively. Then, the two solutions were mixed together and sonicated until the uniform ink was obtained. Then the mixed solution was placed on a heating stir plate at the temperature of 135 ℃ until the DMF completely evaporated. Finally, the obtained powder is subjected to heat treatment in a tubular furnace at 350 ℃ for 2 hours under a H_2/Ar atmosphere to obtain the α-MoC_{1-x}-CoPc@C sample. The CoPc@C sample is obtained using the same method by using C as the substrate.

Materials Characterization:

The crystal structure of the sample was analyzed using an 18 kW advanced X-ray diffraction (XRD) instrument with Cu Kα radiation (λ = 1.54056 Å). The morphology and microstructure of these materials were observed using a JEOL-F200 High Resolution-Transmission Electronic Microscope operating at an accelerating voltage of 200 kV. The energy-dispersive X-ray spectroscopy (EDS) mapping images were captured under the scanning transmission electron microscope (STEM) mode. A double spherical aberration-corrected field emission transmission electron microscope (Titan Themis Z) was used to capture the HAADF-STEM images. X-ray photoelectron spectroscopy (XPS) analysis was conducted using a Mg Kα achromatic X-ray source. The XAFS spectra were obtained at beamline BL14W1 of the Shanghai Synchrotron Radiation Facility (SSRF). Synchrotron-radiation Fourier transform infrared spectroscopy was performed at beamline BL01B of the National Synchrotron Radiation Laboratory (NSRL) For the Co K-edge XAFS measurements, the calculated mass of materials was mixed with carbon black and grid for more than 15 min to ensure the uniformly dispersion. Then, the mixed powders were pressed into a disc for measurements. During XAFS measurements, the adsorption edge energy was firstly calibrated by using Co foil, and the Co K-edge XAFS data was collected by using fluorescence model with Lytle detector. For the C K-edge and Co L-edge XANES measurements, the samples were ground into fine powders and then sticked on the Cu tapes. Then the samples were transferred to the sample chamber for soft X-ray

absorption testing.

Preparation of working electrodes:

The uniformly dispersed catalysts ink was loaded on the carbon paper (CP) to prepare working electrode. CoPc/CP, CoPc@C/CP, α-MoC_{1-x}-CoPc@C/CP were prepared using a typical preparation method. First, 5 mg of catalyst, 750 μl of anhydrous ethanol, 250 μL of DI water and 50 μl of Nafion (5 wt%) were mixed and sonicated for 3h to obtain uniformly dispersed catalyst ink. Then, 100 μl of the above solution was then loaded on carbon paper, the loading amount was keeping at 0.5 mg cm^{-2}.

For CoPc@C/GDL and α-MoC_{1-x}-CoPc@C/GDL, 10 mg of the synthesized CoPc@C and α-MoC_{1-x}-CoPc@C powders were dispersed in 2 mL of anhydrous ethanol containing 30 μL Nafion (5 wt%). Then, the mixtures were ultrasonically dispersed for 3 h to prepare catalyst ink. Each catalyst ink was deposited on a 2*2 cm GDL using a pipette gun. The loading of CoPc@C and α-MoC_{1-x}-CoPc@C catalysts on the electrode was approximately 0.5 mg cm^{-2}.

Electrochemical measurements:

For H-Cell testing:

The CO_2 electroreduction reaction was performed in a typical three-electrode electrochemical cell. The cathodic and anodic compartments were separated by a proton exchange membrane (Nafion 117). Both compartments were filled with CO_2-saturated 0.5 M $KHCO_3$ solution. The reference and counter electrodes were an Ag/AgCl (3.5 M KCl) electrode and a Pt foil, respectively. Prior to each experiment, pure CO_2 gas (99.999 %) was bubbled into the electrolyte for 30 minutes to ensure the CO_2 saturation. During electrolysis, the electrolyte in the cathodic compartment was stirred at a rate of 200 rpm. Linear sweep voltammetry (LSV) curves were conducted at a scanning rate of 5 mV s^{-1} from 0 to -1.2 V (versus RHE). During the chronoamperometry, CO_2 was continuously flowed into the electrolyte at a rate of 20 sccm.

For flow cell testing:

The entire flow cell setup comprises a liquid circulation system, a flow cell and a

gas input system. The flow cell consists of a gas flow chamber, a catholyte chamber and an anolyte chamber with a reaction area of 1 cm ². The cathode GDL catalyst faces the cathode liquid chamber, with the substrate facing the gas flow chamber, while a nickel foam serves as the counter electrode in the anode chamber. The cathode liquid chamber and anode liquid chamber are separated by an anion exchange membrane (FAA-3-PK-130, Fumasep). The reference electrode, Hg/HgO (1 M KOH), is positioned within the cathode liquid chamber. During the assembly of the flow cell, a torque wrench is used to ensure equal torque on the four bolts. The liquid circulation system incorporates two peristaltic pumps, with the liquid flowing into the chambers from the bottom and out from the top. The flow rates connecting the cathode liquid chamber and the anode liquid chamber are set at 20 mL min^{-1} and 50 mL min^{-1}, respectively. Additionally, the CO_2 input flow rate into the gas flow chamber is regulated at 20 mL min^{-1} using a flow meter (D08-1F, manufactured by Beijing Sevenstar Flow Co., Ltd.). All potentials were converted to RHE via the equation as follows:

$$E\ (RHE) = E\ (Ag/AgCl) + 0.059 \times pH + 0.197\ V$$

$$E\ (RHE) = E\ (Hg/HgO) + 0.059 \times pH + 0.098\ V$$

Unless otherwise stated, all potentials are referenced to the standard hydrogen electrode.

The gas products were analyzed using a gas chromatograph (GC9790 Plus) equipped with a flame ionization detector (FID) and a thermal conductivity detector (TCD). The TCD was primarily used for quantifying H_2, while the FID was mainly used for quantifying CO. The Faradaic efficiency for CO was calculated using the following formula:

$$FE_{CO} = \frac{nFV}{Itotal} \times 100\%$$

In this formula, *n* represents the number of electrons transferred for the product, *F* is the Faraday constant (96485 C mol^{-1}), *V* denotes the molar flow rate of the product, and I_{total} is the total electrolytic current density. The calculation of the partial current density for CO is as follows:

$$J_{CO} = I_{total} \times FE_{CO}$$

Electrochemical impedance spectroscopy (EIS):

This study employed an electrochemical workstation equipped with a three-electrode setup. All impedance measurements for EIS were conducted at -0.53 V versus RHE, with a measurement interval of 0.05 V. The frequency range spanned from 100,000 Hz to 0.1 Hz, with an AC amplitude of 5 mV. The obtained EIS data were fitted with circuit models using the Zview2 Analyzer.

In-situ XAFS measurement:

The in-situ XAFS measurement was tested in a home-made in-situ electrochemical cell by using fluorescence acquisition mode through Lytle detector. The catalyst ink was prepared by uniformly disperse 20 mg catalyst in 1 mL ethanol solution containing 50 μL Nafion (5 wt%) followed by sonication for 1 h. Then the catalyst ink was dropped onto the GDL with a catalyst loading of 1 mg cm^{-2} and dried in a vacuum oven overnight. During in-situ test, the prepared GDL was assembled in electrochemical cell sealed with Kapton tape. The CO_2-saturated 0.5 M $KHCO_3$ solution was using as the electrolyte while the Ag/AgCl and Pt mesh serve as the reference and counter electrodes. The CO_2 was continuous blowing into the electrolyte with a flow rate of 20 sccm to ensure the CO_2-saturated environment. During the in-situ CO_2RR process, the XAFS data under different potentials was collected for further analysis. The XAFS data was analyzed by ATHENA and ARTEMIS software. The background signals of absorption curves were firstly deducted and the then normalized to 1. The FT-EXAFS curves, which reflect the local configuration in R space, were obtained by Fourier transformed χ(k) function using Hanning window in the range of 2.7 to 11.6 $Å^{-1}$ (d_k=1.0 $Å^{-1}$). The FT-EXAFS data was fitted by ARTEMIS software by using CoPc as references: Firstly, the amplitude reduction factors (S_0^2) was obtained by fitting the CoPc with fixed coordination number; Secondly, the local coordination information of Co by fitting the FT-EXAFS spectra using obtained amplitude reduction factors and different structure parameter until the fitting data is highly consistent with experimental data.

In-situ ATR-SEIRAS measurement:

In situ ATR-SEIRAS measurement was performed at the infrared beamline BL01B of the HLS through a homemade top-plate cell-reflection infrared set-up with a ZnSe crystal as the infrared transmission window (cut-off energy of ~625 cm^{-1}). This end station was equipped with an FTIR spectrometer (Bruker 70 v/s) with a KBr beam splitter and various detectors (herein, a liquid-nitrogen-cooled mercury cadmium telluride detector was used) coupled with an infrared microscope (Bruker Hyperion 2000) with an ×15 objective. The catalyst electrode was tightly pressed against the ZnSe crystal window with a micrometre-scale gap to reduce the loss of infrared light. To ensure the quality of spectra, the apparatus adopted a reflection mode with a vertical incidence of infrared light. Each infrared absorption spectrum was acquired by averaging 128 scans at a resolution of 4 cm^{-1}. The background spectrum of the catalyst electrode was acquired at an open-circuit voltage before each systemic measurement, and the measured potential range was −0.5 to −1.1 V_{RHE}. All the electrochemical tests were performed in CO_2-saturated 0.5 M $KHCO_3$ electrolyte. The sample loading amount of each test was 40 μg (20 μL). The measurements were conducted using a custom-built three-electrode electrochemical cell. A platinum wire was used as the counter electrode, and a saturated Ag/AgCl electrode served as the reference electrode. The catalyst was deposited on an Au-coated silicon prism for analysis. All spectra were collected by averaging 64 scans at a resolution of 4 cm^{-1} using a Bruker Vertex 80V spectrometer equipped with a liquid nitrogen-cooled mercury cadmium telluride (LN-MCT) detector. The background spectrum was recorded under open-circuit voltage conditions, while subsequent spectra were obtained by applying voltage in stepwise increments.

Theoretical calculations.

It should be noted that DFT+U method can underestimate the adsorption energy, we used DFT method to examine the adsorption free energy profiles of CO_2RR and HER [1, 2]. All density functional theory (DFT) calculations are conducted by using Vienna ab initio simulation package (VASP) [3, 4], with generalized gradient approximation (GGA) in Perdew, Burke, and Ernzerhof (PBE) functional [5], and projector augmented wave (PAW) method [6, 7]. As to the effect of

molecule-substrate interaction, the DFT-D2 method with van der Waals force correction is used. The plane-waved energy cutoff is set to be 500 eV. The convergence of force between atoms for optimization is set to 0.02 eV/Å, and the convergence of total energy for wave function self-consistent are set to 10^{-5} eV. The vacuum size is chosen as 20 Å to avoid interaction between two slabs for all structures. The climbing-image nudged elastic band (CINEB) algorithm is used to calculate the transition-state energies of water dissociation step. The CINEB algorithm was run until the maximum force was less than 0.04 eV/Å. To simulate the ice-like water on the α-MoC_{1-x} surface, the top water-layer of hydrogen bonding network and the bottom atom-layer of α-MoC_{1-x} are fixed, while the other are fully relaxed.

The Gibbs free energy change (ΔG) is written as below [8]:

$\Delta G = \Delta E + \Delta ZPE - T\Delta S + \Delta G_U$

Where the initial and final state involving an electron in the electrode is considered by shifting the energy of the state by $\Delta G_U = -neU$, and U is the electrode applied potential relative to reversible hydrogen electrode (RHE).

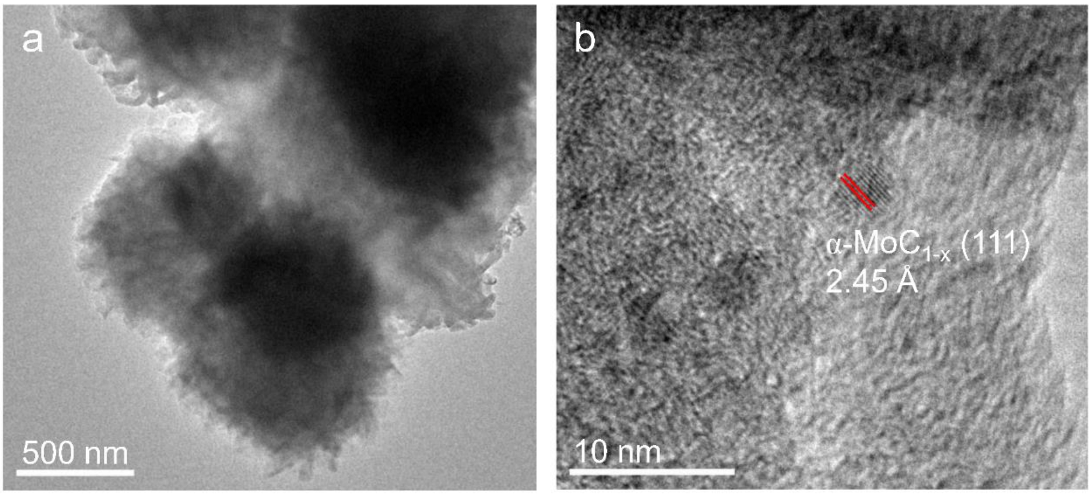

Figure S1. (a) TEM and (b) HRTEM images of α-MoC_{1-x}@C.

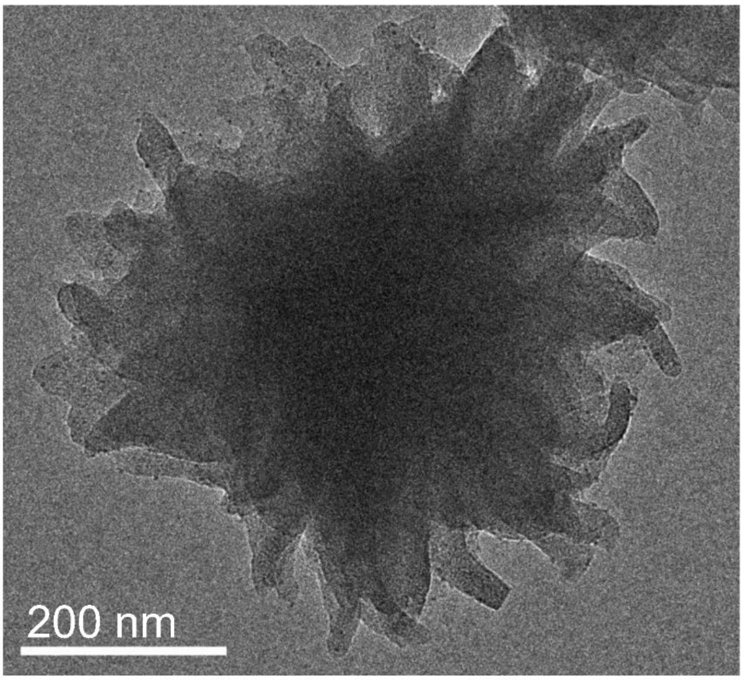

Figure S2. TEM image of α-MoC_{1-x}-CoPc@C.

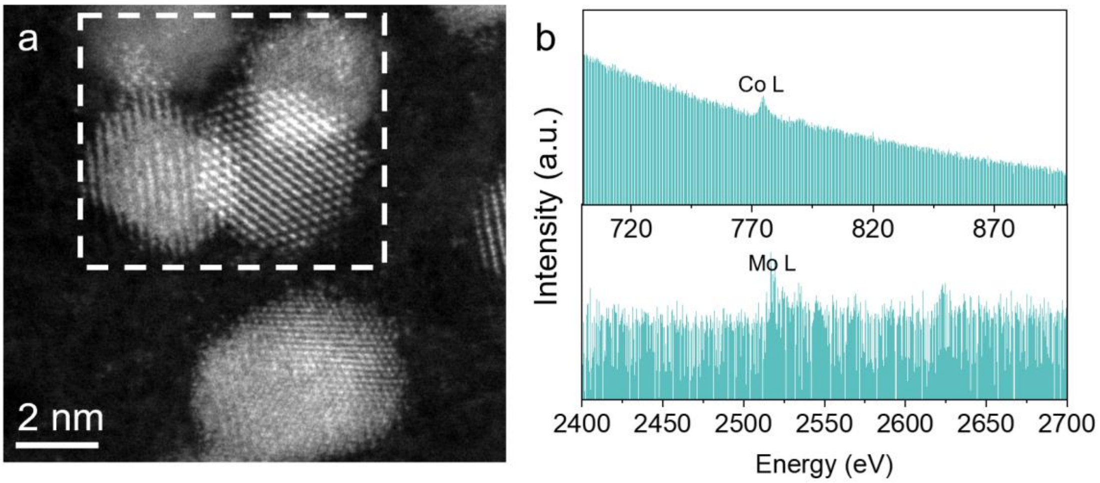

Figure S3. (a) HAADF-STEM image of the α-MoC_{1-x}-CoPc@C and (b) the corresponding EELS spectra of the pointed white dashed line zone in (a).

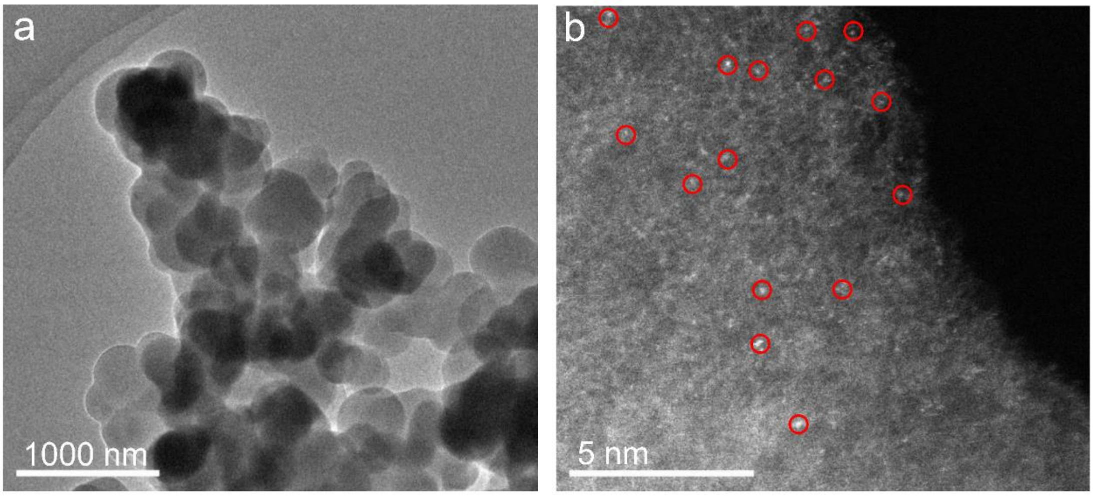

Figure S4. (a) TEM and (b) HAADF-STEM images of CoPc@C.

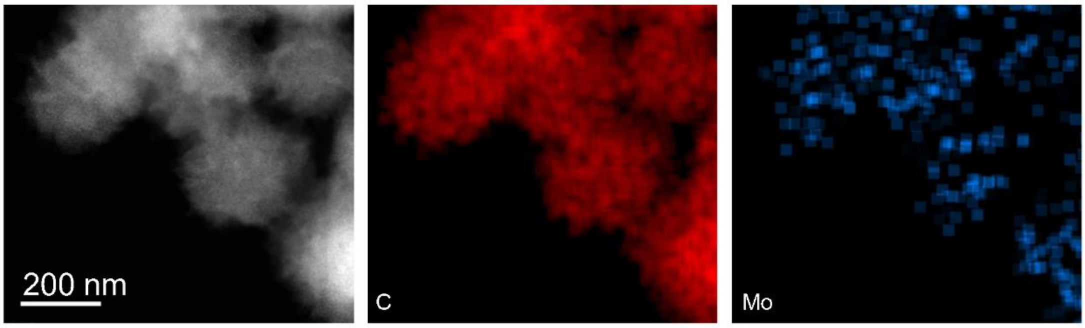

Figure S5. Elemental mapping images of α-MoC_{1-x}@C. (Note: the elemental -mapping images were collected on the FEI Themis Z 300 kV Double spherical aberration correction TEM equipped with four detector probe).

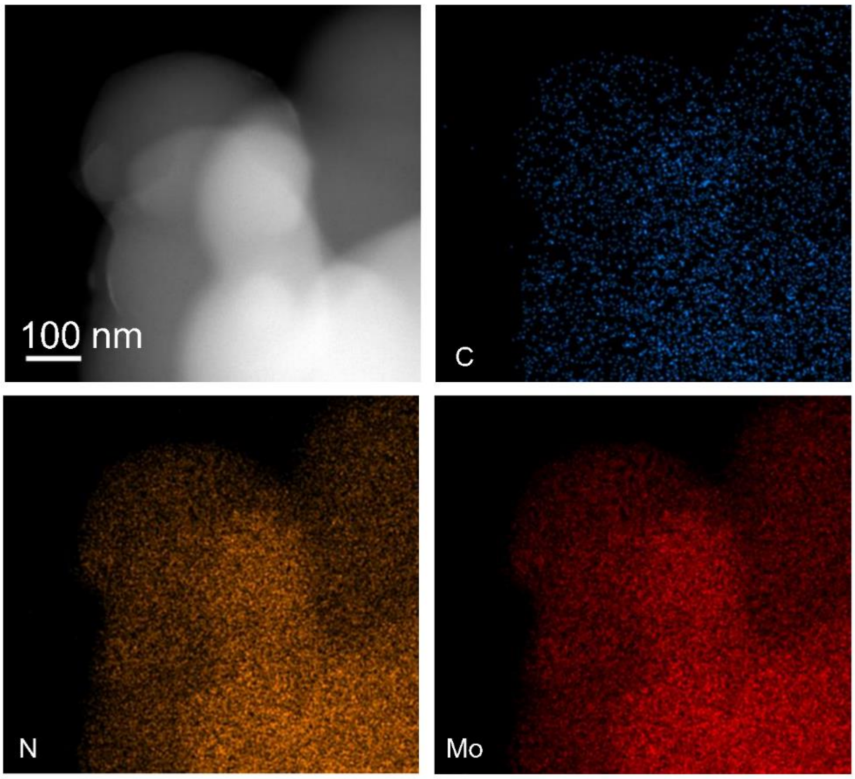

Figure S6. Elemental mapping images of CoPc@C. (Note: the elemental mapping images were collected on the JEOL F200 TEM equipped with single detector probe).

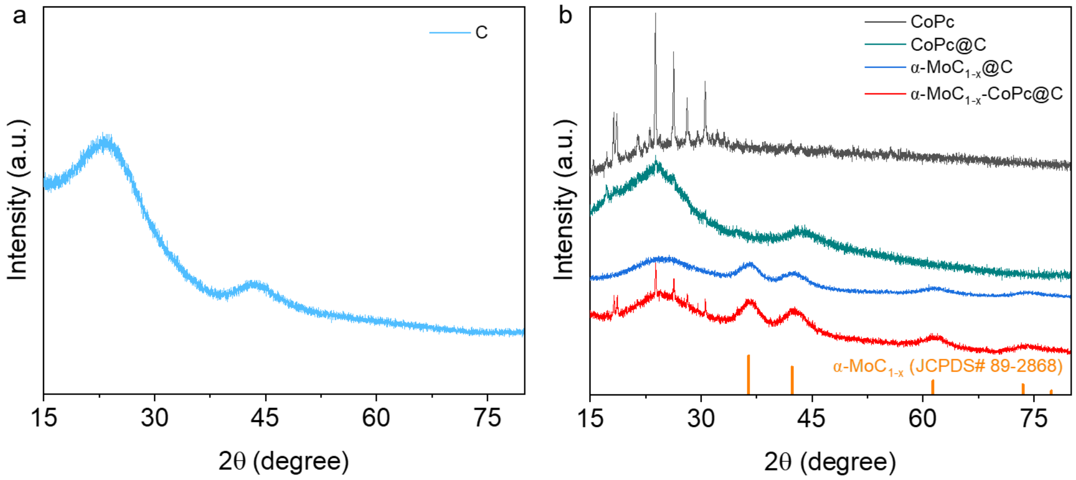

Figure S7. XRD patterns of (a) C and (b) other samples. Note: the XRD pattern of C is significantly high due to the strong diffraction, hence the XRD pattern of C is separate listed in Figure S6a.

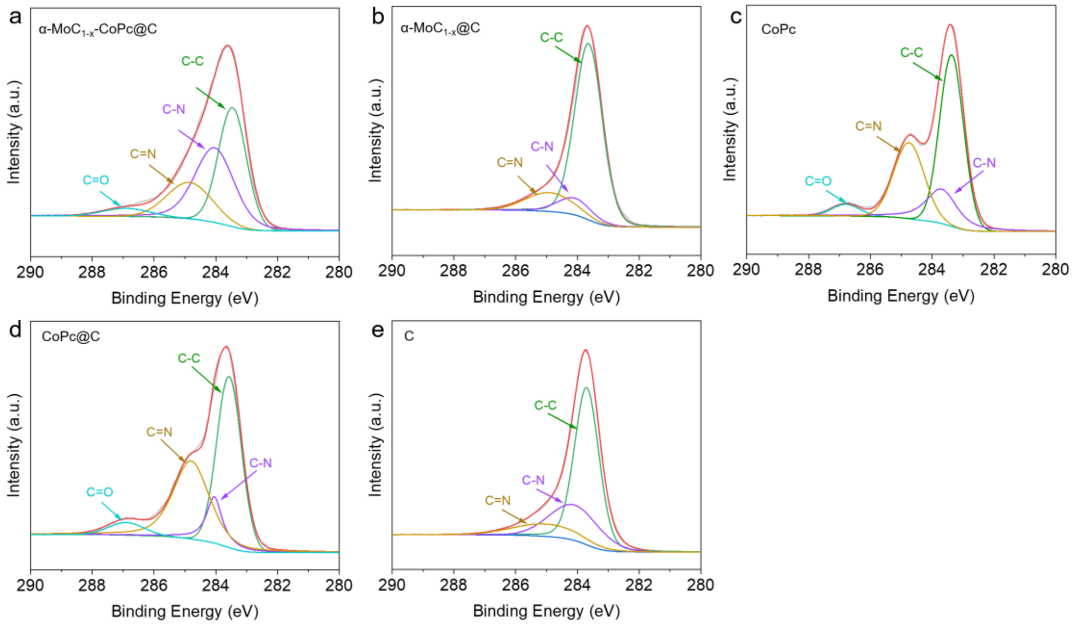

Figure S8. C 1s XPS spectra and corresponding fitting results of (a) α-MoC_{1-x}-CoPc@C, (b) α-MoC_{1-x}@C, (c) CoPc, (d) CoPc@C and (e) C.

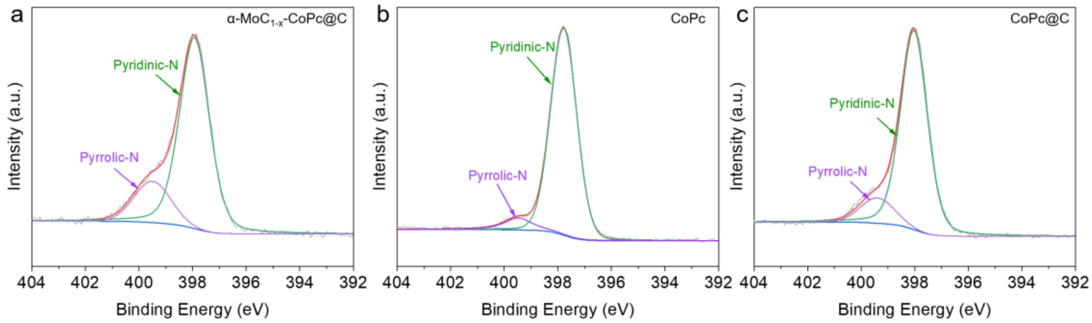

Figure S9. N 1sXPS spectra and corresponding fitting results of (a) α-MoC_{1-x}-CoPc@C, (b) CoPc, (c) CoPc@C@C.

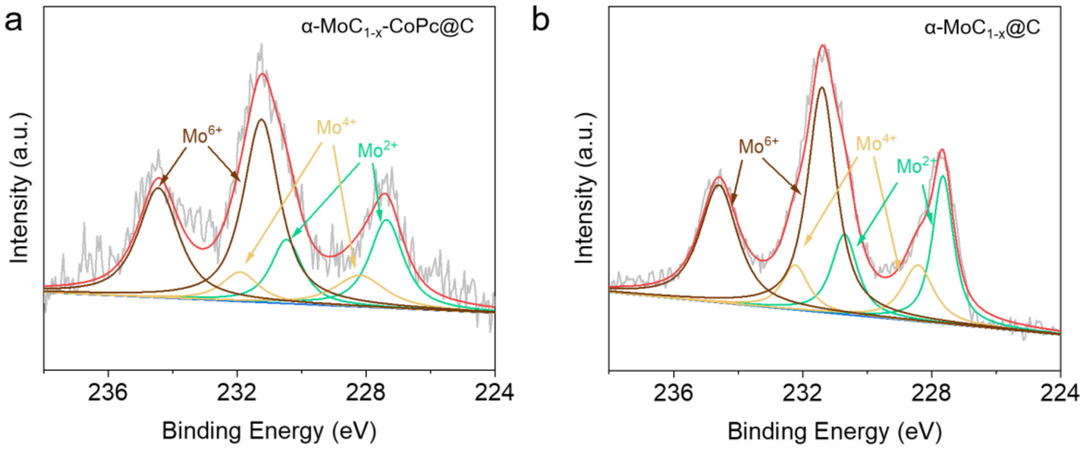

Figure S10. Mo 3d XPS spectra of (a) α-MoC_{1-x}-CoPc@C, and (b) α-MoC_{1-x}@C.

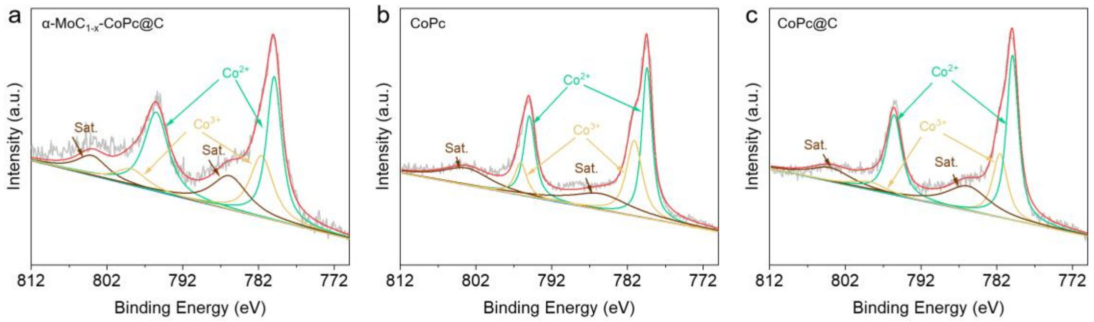

Figure S11. Co 2p XPS spectra of and corresponding fitting results of (a) α-MoC_{1-x}-CoPc@C, (b) CoPc, and (c) CoPc@C.

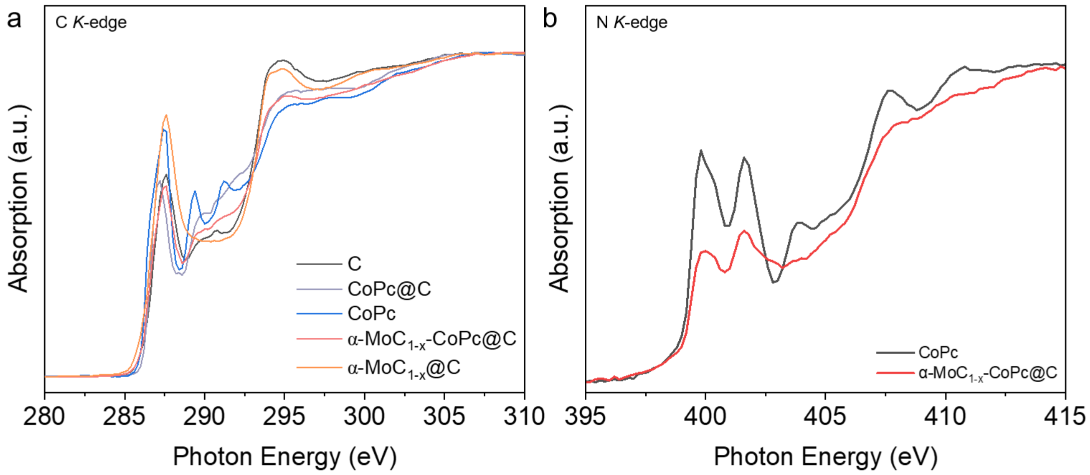

Figure S12. (a) C K-edge XANES spectra of α-MoC_{1-x}-CoPc@C and counterparts. (b) N K-edge XANES spectra of α-MoC_{1-x}-CoPc@C and CoPc.

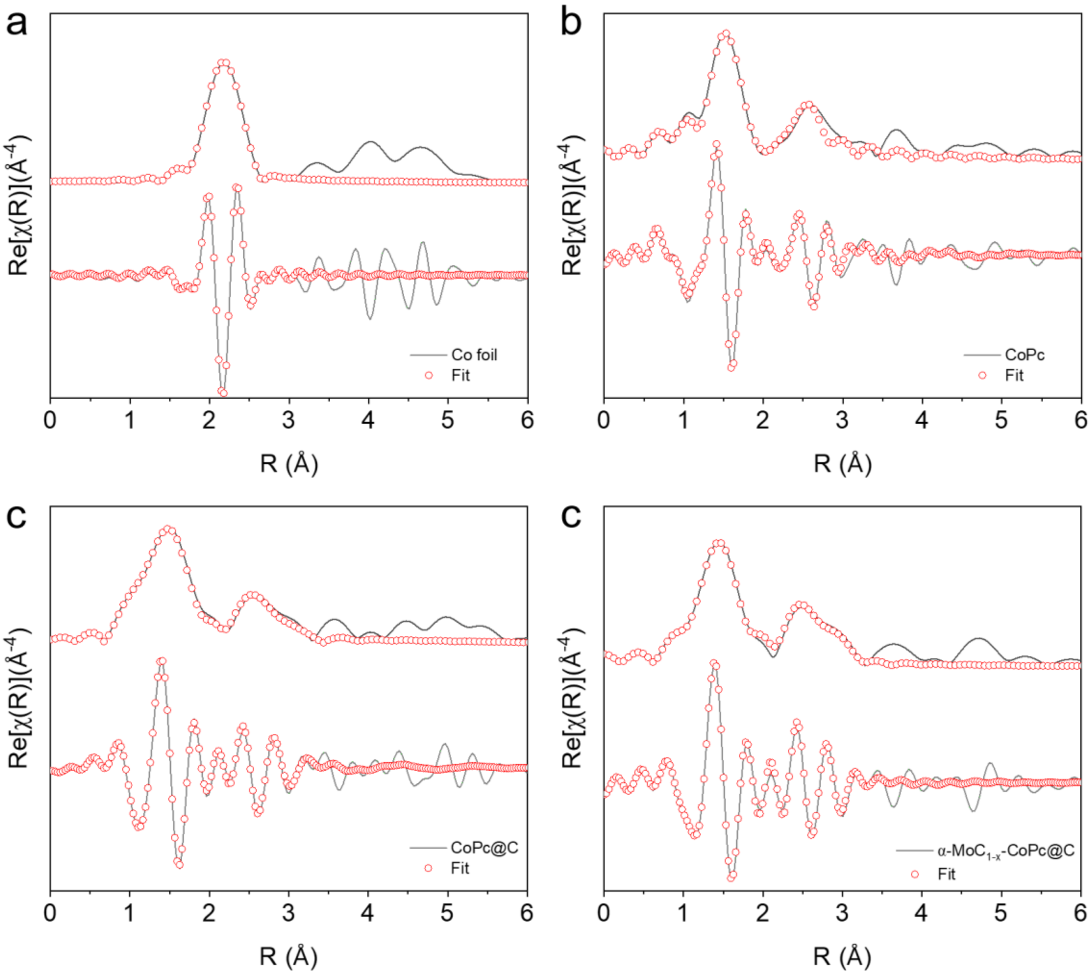

Figure S13. EXAFS fitting curves displayed in R space for (a) Co foil, (b) CoPc, and (c) CoPc@C, and (d) α-MoC_{1-x}-CoPc@C.

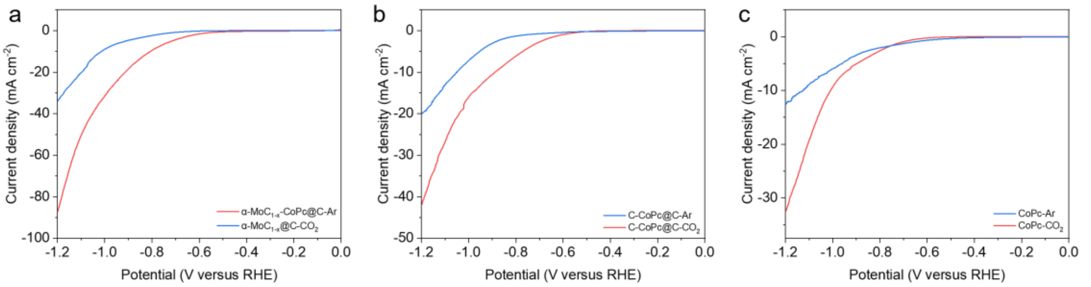

Figure S14. LSV curves of (a) α-MoC_{1-x}-CoPc, (b) CoPc@C, and (c) CoPc under CO_2 and Ar saturated electrolyte in H-cell.

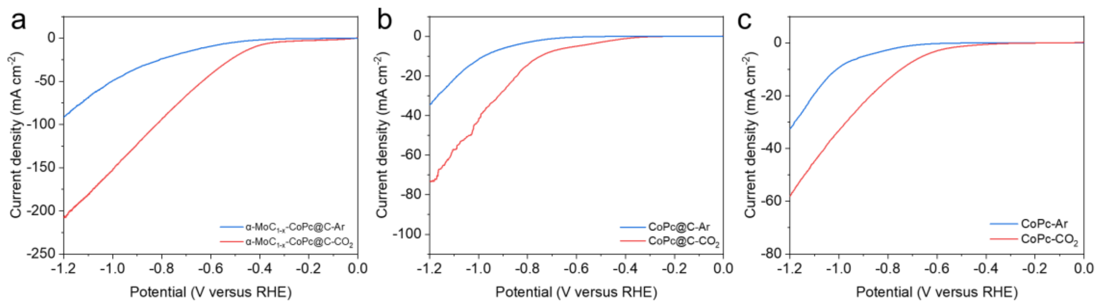

Figure S15. LSV curves of (a) α-MoC_{1-x}-CoPc@C, (b) CoPc@C, and (c) CoPc under CO_2 and Ar saturated electrolyte in flow-cell.

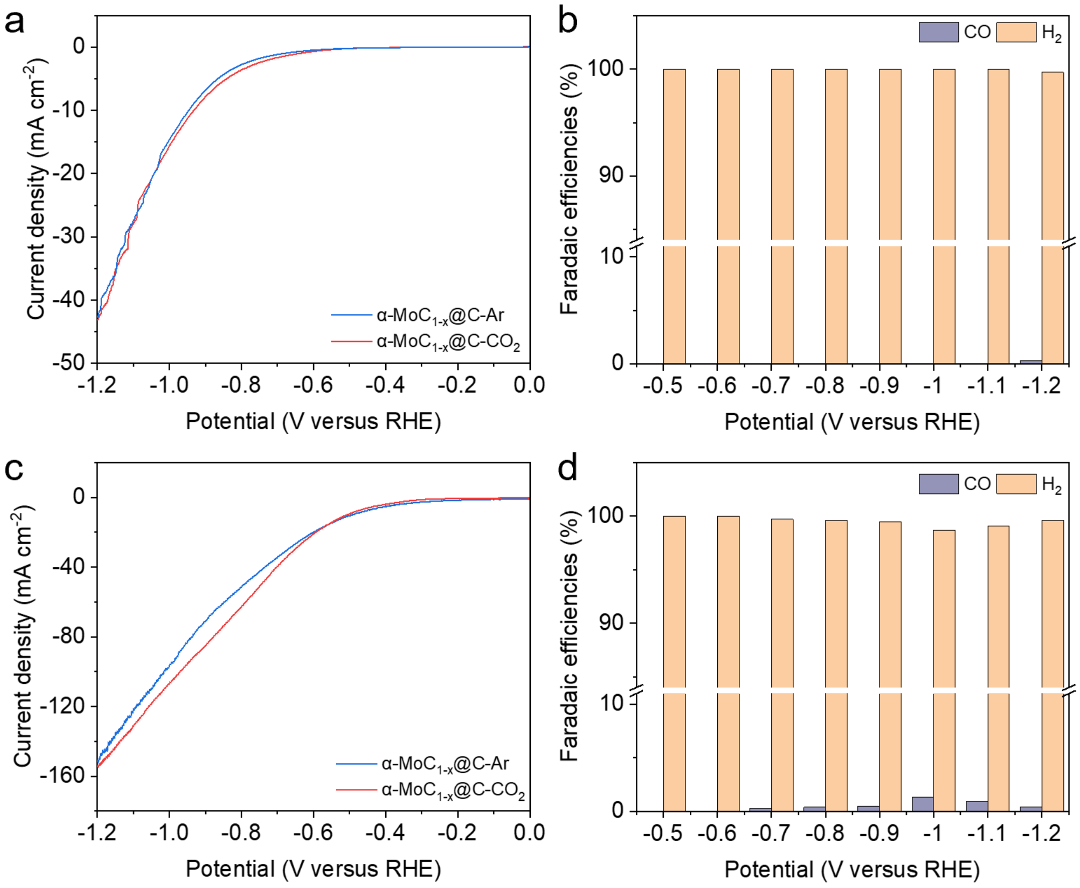

Figure S16. LSV curves of the α-MoC_{1-x}@C under CO_2 and Ar as feeding gas for (a) H-cell and (c) flow-cell assembly. (b) and (d) are the corresponding Faradaic efficiencies of the H-cell and flow-cell measurements.

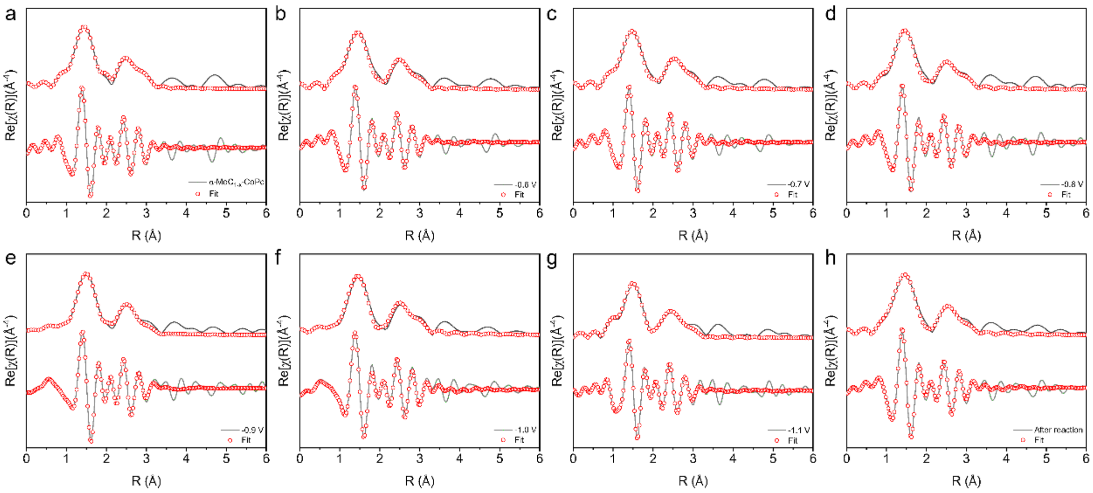

Figure S17. EXAFS fitting curves displayed in R space for α-MoC_{1-x}-CoPc@C under different working state during operando measurement.

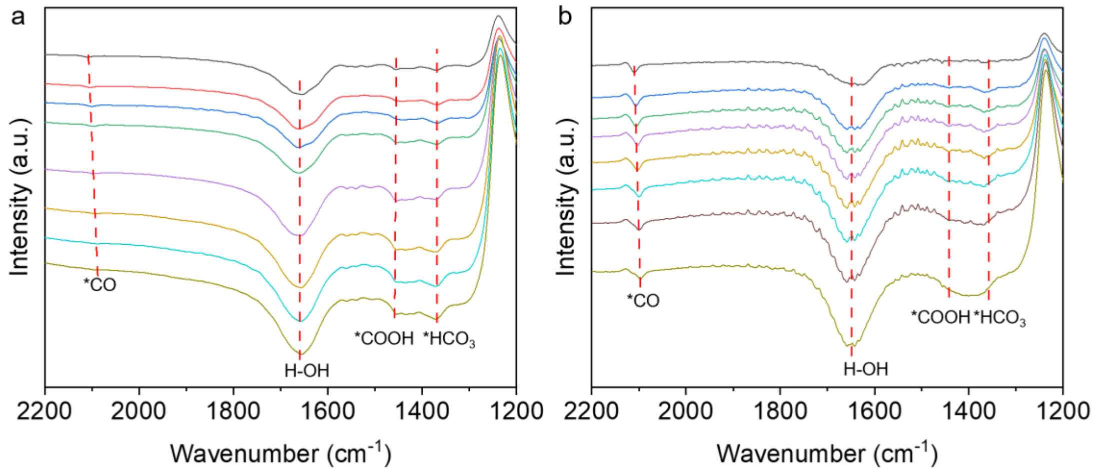

Figure S18. ATR-SEIRAS spectra of (a) α-MoC_{1-x}-CoPc@C and (b) CoPc@C at the wavenumber range from 1200 cm^{-1} to 2200 cm^{-1} under different applied potentials.

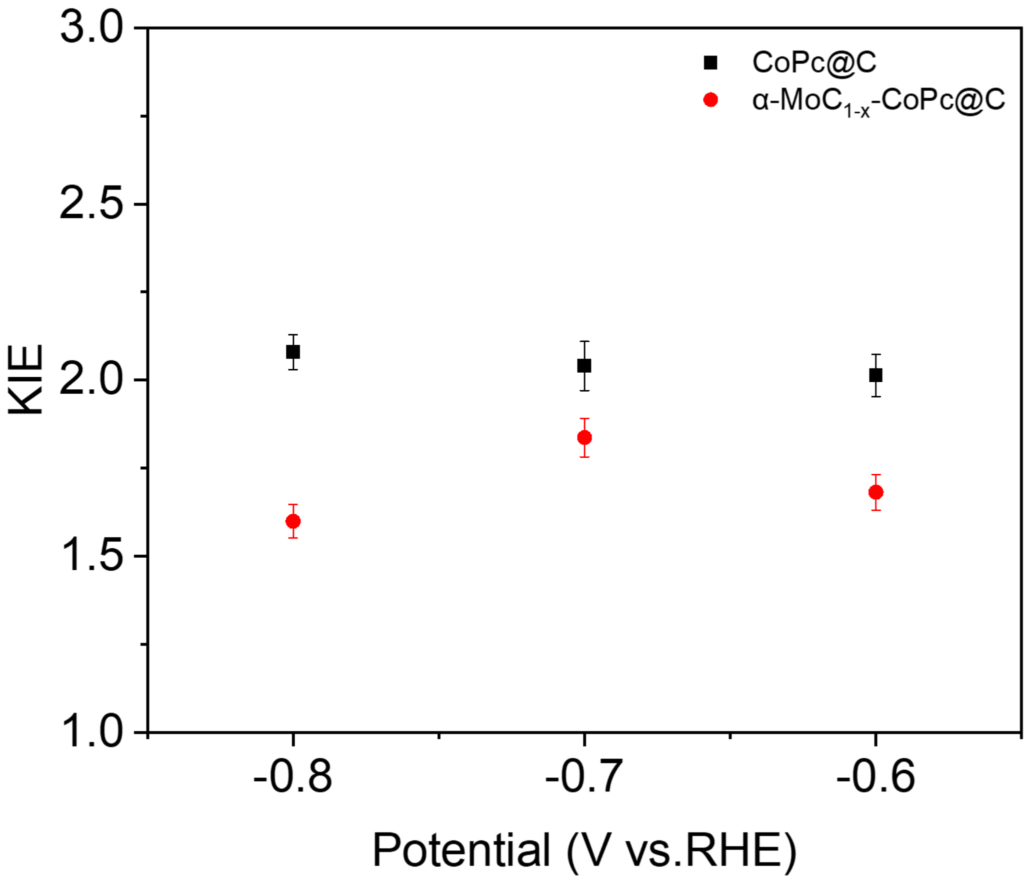

Figure S19. KIE values of α-MoC_{1-x}-CoPc@C and CoPc@C at different potentials.

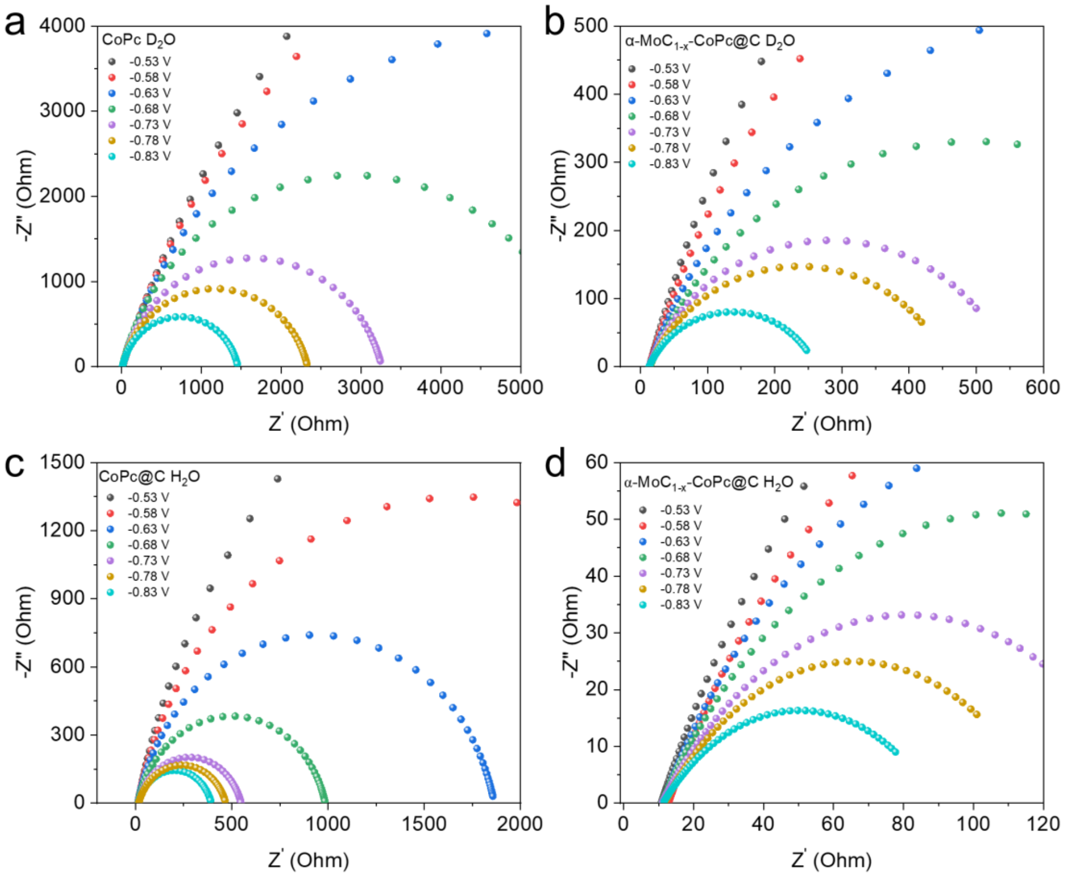

Figure S20. Nyquist plots under different potentials of α-MoC_{1-x}-CoPc@C and CoPc@C by using D_2O and H_2O as electrolyte.

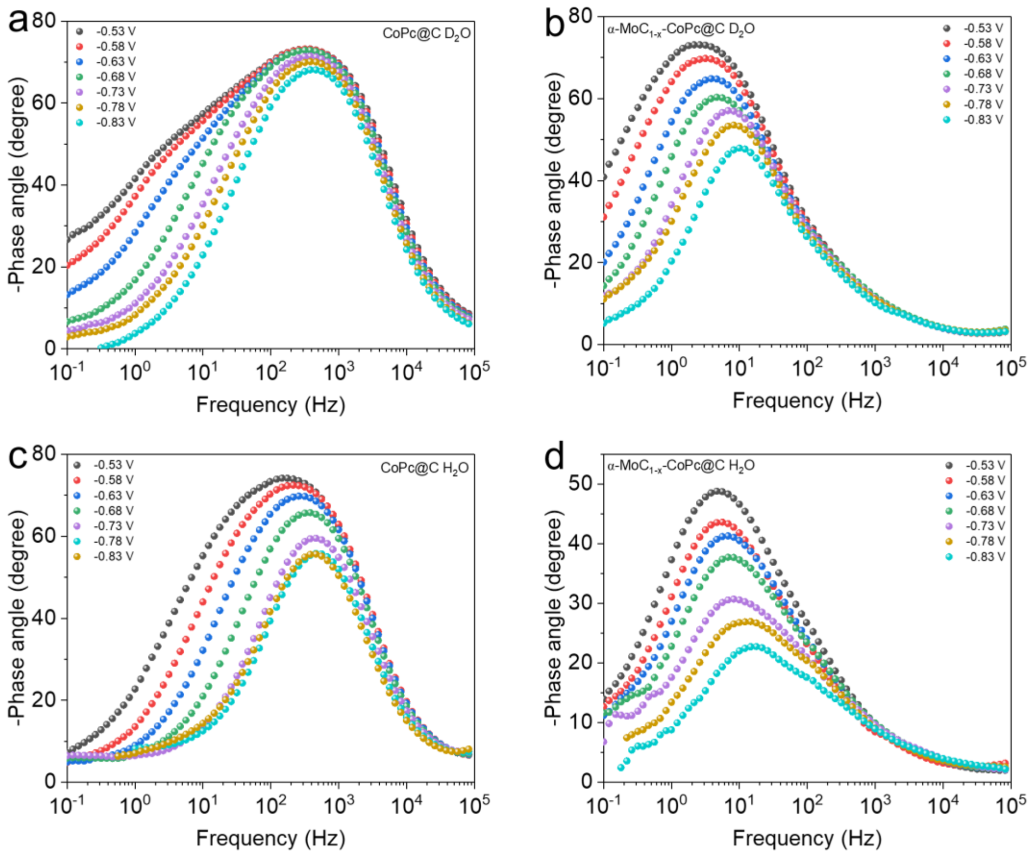

Figure S21. Bode plots of under different potentials of α-MoC_{1-x}-CoPc@C and CoPc@C by using D_2O and H_2O as electrolyte.

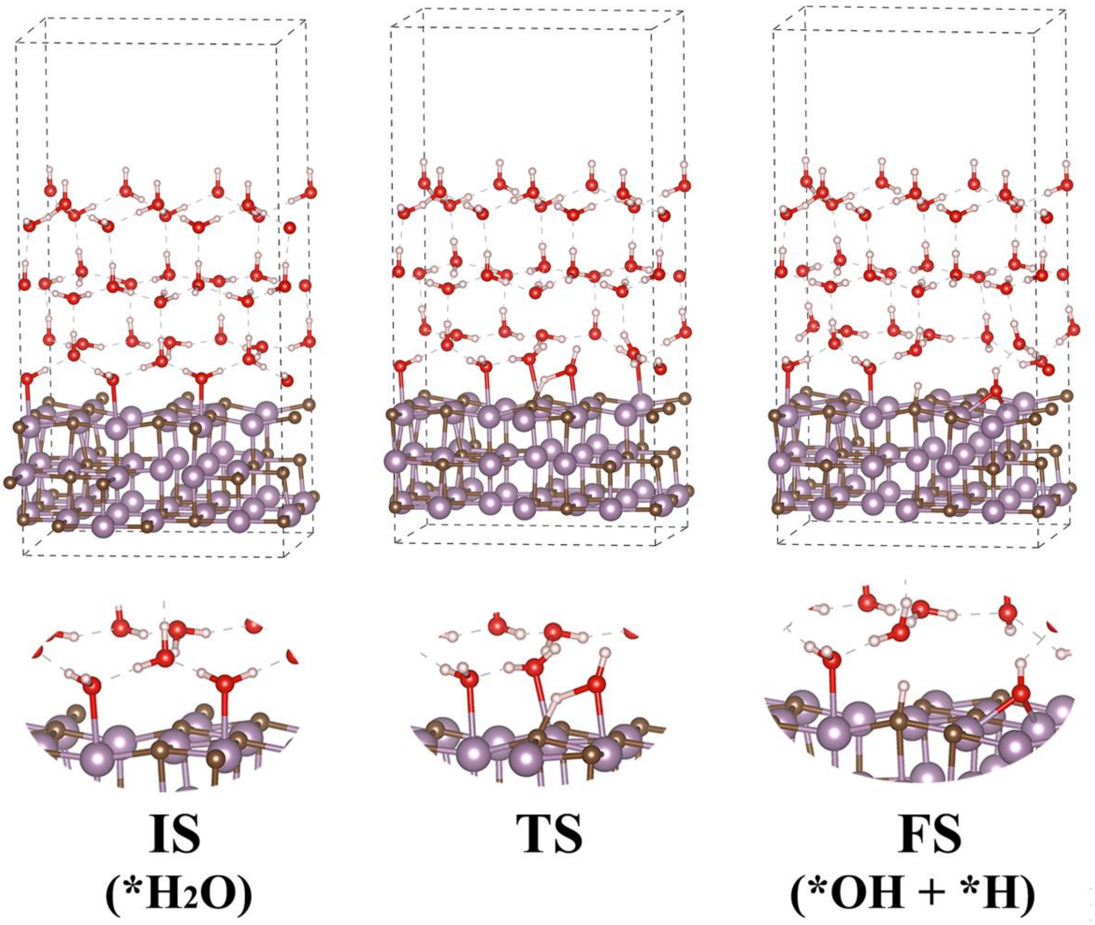

Figure S22. The atomic structures of initial state (IS), transition state (TS), and final state (FS) of the elementary reaction step of H_2O dissociation into adsorbed *OH and *H (* + $H_2O \rightarrow$ *OH + *H).

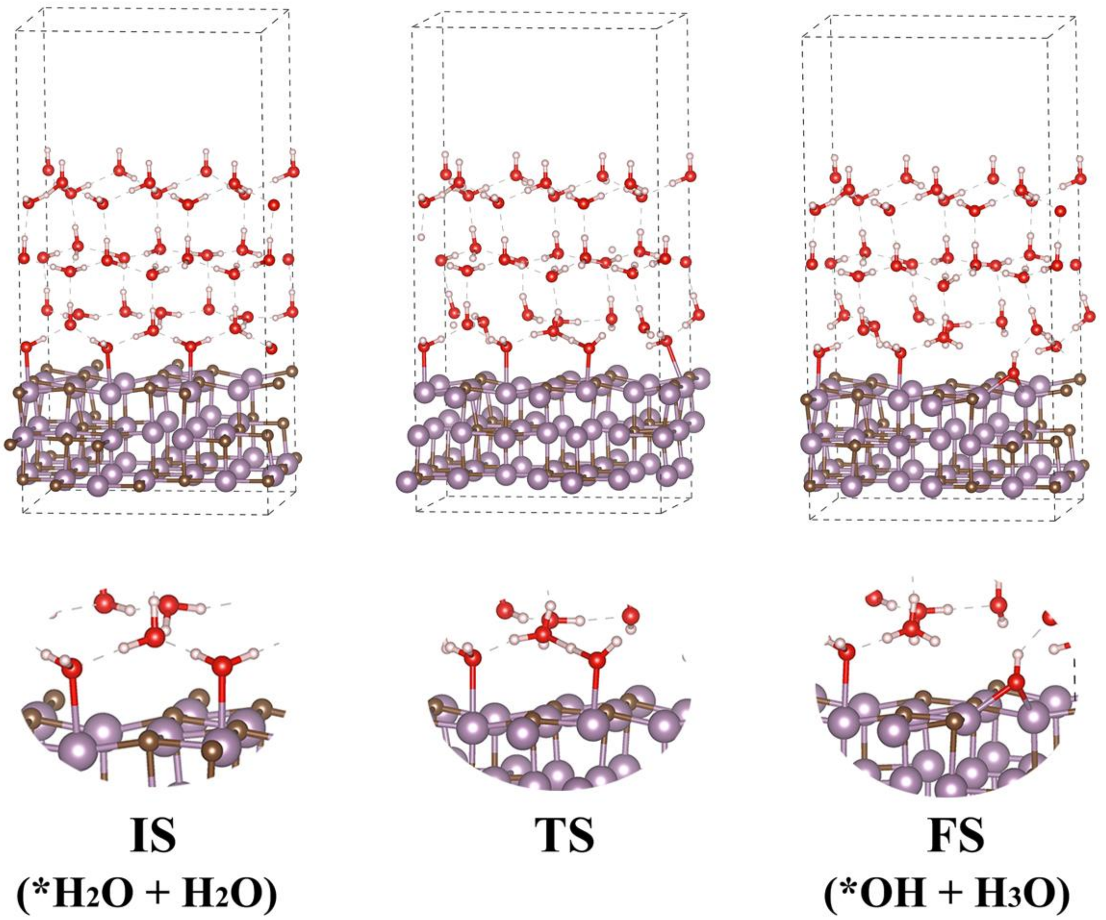

Figure S23. The atomic structures of initial state (IS), transition state (TS), and final state (FS) of the elementary reaction step of H_2O dissociation into adsorbed *OH and proton staying in the hydrogen bond network (* + H_2O⟶*OH + H_3O).

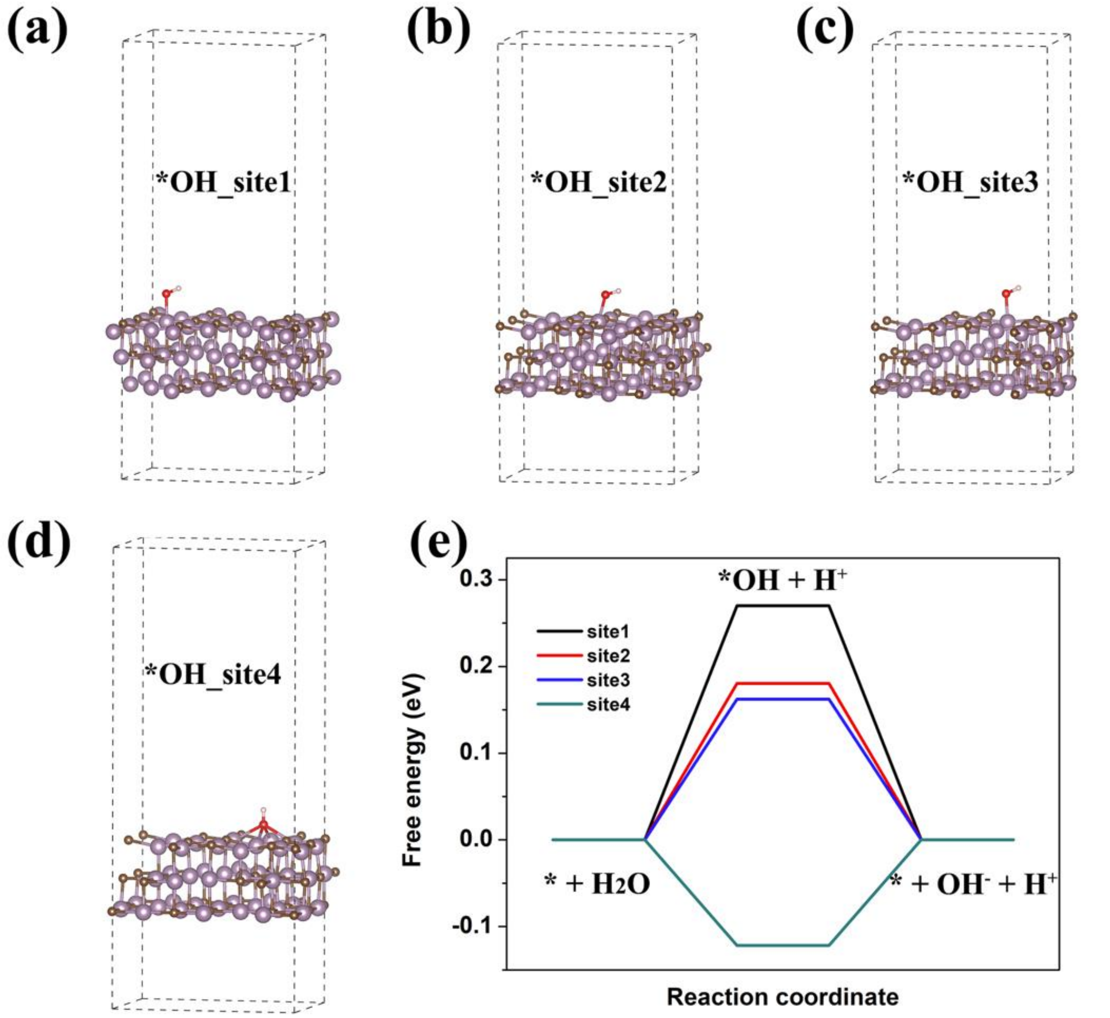

Figure S24. The four adsorption sites of *H on the α-MoC_{1-x} surface (a, b, c, d), and the corresponding free energy profiles of H2O dissociation into hydroxyl ion and proton on these four adsorption sites (e).

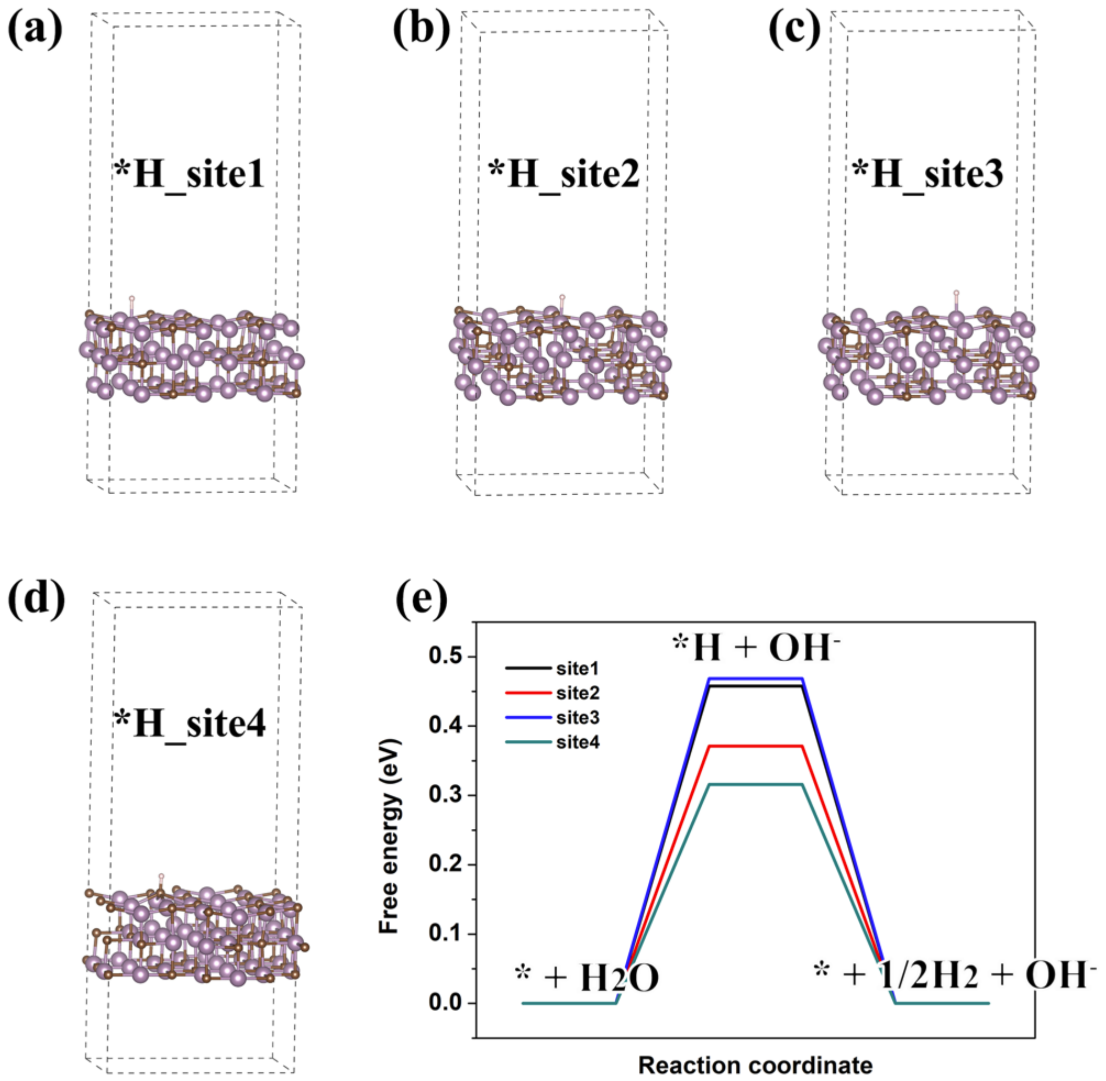

Figure S25. The four adsorption sites of *OH on the α-MoC_{1-x} surface (a, b, c, d), and the corresponding free energy profiles of hydrogen evolution reaction (HER) with H_2O as reactant on these four adsorption sites (e).

Table S1. Mass loading of the Mo and Co atoms for the as-prepared samples.

Elements / wt% / Materials	Mo	Co
α-MoC_{1-x}-CoPc@C	9.89	0.99
α-MoC_{1-x}@C	9.64	
CoPc@C		1.11

Table S2. EXAFS fitting results of Co K-edge in R space

sample	Scattering pair	CN	R(Å)	$\sigma^2(10^{-3}Å^2)$	ΔE_0 (eV)	R factor
Co foil	Co-Co	12.00	2.49	6.18	7.04	0.002
CoPc	Co-N	4.00	1.91	3.29	8.53	0.02
	Co-C	8.00	2.94	3.70	9.28	
	Co-C/N	16.00	3.13	3.70	8.52	
CoPc@C	Co-N	4.02	1.91	2.88	6.72	0.02
	Co-C	8.00	2.94	4.23	7.88	
	Co-C/N	16.03	3.13	4.23	7.88	
α-MoC_{1-x}-CoPc	Co-N	4.56	1.88	4.87	2.96	0.004
	Co-C	8.52	2.94	3.03	7.31	
	Co-C/N	16.22	3.12	0.12	8.99	
α-MoC_{1-x}-CoPc@-0.6 V	Co-N	4.23	1.89	4.62	5.11	0.01
	Co-C	7.80	2.95	2.47	8.85	
	Co-C/N	13.63	3.15	3.81	9.77	
α-MoC_{1-x}-CoPc@-0.7 V	Co-N	4.32	1.90	4.37	5.44	0.01
	Co-C	7.70	2.95	2.25	9.11	
	Co-C/N	12.69	3.14	0.03	9.35	
α-MoC_{1-x}-CoPc@-0.8 V	Co-N	4.51	1.90	4.86	4.75	0.02
	Co-C	7.52	2.95	2.23	9.11	
	Co-C/N	13.16	3.13	1.65	9.35	
α-MoC_{1-x}-CoPc@-0.9 V	Co-N	4.49	1.89	5.51	3.01	0.02
	Co-C	7.33	2.94	2.64	7.11	
	Co-C/N	14.38	3.16	6.94	9.55	
α-MoC_{1-x}-CoPc@-1.0 V	Co-N	4.04	1.89	4.92	4.92	0.01
	Co-C	7.52	2.94	2.45	7.23	
	Co-C/N	10.52	3.16	0.32	9.22	
α-MoC_{1-x}-CoPc@-1.1 V	Co-N	4.23	1.89	4.26	3.78	0.01
	Co-C	7.70	2.92	2.62	5.81	
	Co-C/N	10.24	3.16	1.11	9.87	
α-MoC_{1-x}-CoPc@after bias	Co-N	3.94	1.91	2.97	8.18	0.01
	Co-C	8.46	2.95	4.00	8.07	
	Co-C/N	13.16	3.17	2.76	8.57	

Here, CN represents the coordination number, R represents the bond length, σ^2 represents the Debye-Waller factor value. S_0^2 was determined from standard reference fitting.

Table S3. EIS fittings parameters of α-MoC_{1-x}-CoPc by using H_2O containing electrolyte.

**Potential** (v)	**R_s(Ω)**	**R_1(Ω)**	**CPE_1-T** $S \cdot s^n \cdot cm^{-2}$	**CPE_1-P**
-0.53	10.74	430.6	0.0011356	0.68887
-0.58	12.76	304.6	0.0012798	0.67261
-0.63	11.14	237	0.0014361	0.65362
-0.68	10.87	196	0.001682	0.61178
-0.73	10.89	140.5	0.0021233	0.56171
-0.78	11.64	108.2	0.0020906	0.55033
-0.83	11.2	78.24	0.00015942	0.50358

Note: n=CPE-P.

Table S4. EIS fittings parameters of α-MoC_{1-x}-CoPc by using D_2O containing electrolyte.

**Potential** (v)	**R_s(Ω)**	**R_1(Ω)**	**CPE_1-T** $S \cdot s^n \cdot cm^{-2}$	**CPE_1-P**
-0.53	13.6	5125	0.00037684	0.83068
-0.58	13.61	2893	0.00041543	0.8139
-0.63	13.78	1508	0.00046554	0.78976
-0.68	14.32	963.9	0.00050541	0.7657
-0.73	13.12	538	0.000536	0.76875
-0.78	13.71	446.2	0.00059802	0.74327
-0.83	13.01	248.5	0.00066655	0.73121

Note: n=CPE-P.

Table S5. EIS fittings parameters of CoPc@C by using H_2O containing electrolyte.

Potential (v)	$R_s(\Omega)$	$R_1(\Omega)$	CPE_1-T $S \cdot s^n \cdot cm^{-2}$	CPE_1-P
-0.53	12.47	6288	1.369E-05	0.86073
-0.58	12.52	3351	1.3107E-05	0.86293
-0.63	12.8	1851	1.3645E-05	0.85953
-0.68	13.16	972.6	1.514E-05	0.85008
-0.73	14.09	534.4	1.931E-05	0.82433
-0.78	16.28	452.7	2.2726E-05	0.81155
-0.83	15.1	378	1.9055E-05	0.8305

Note: n=CPE-P.

Table S6. EIS fittings parameters of CoPc@C by using D_2O containing electrolyte.

Potential (v)	$R_s(\Omega)$	$R_1(\Omega)$	CPE_1-T $S \cdot s^n \cdot cm^{-2}$	CPE_1-P
-0.53	11.9	26831	1.2808E-05	0.66417
-0.58	18216	47232	1.232E-05	0.65559
-0.63	10731	15977	1.1702E-05	0.65064
-0.68	5760	4583	9.2924E-06	0.69226
-0.73	12.94	3247	9.69E-06	0.76724
-0.78	12.9	2319	1.0028E-05	0.7946
-0.83	13.64	1445	9.1233E-06	0.76674

Note: n=CPE-P.

References

[1] Y, Wang, Y. Tang, *J. Am. Chem. Soc.* **2019**, 141, 14115–14119.
[2] Z. Zhang, et al., *Angew. Chem. Int. Ed.*, **2018**, 57, 16339-16342.
[3] G. Kresse, J. Hafner, *Phys. Rev. B* **1993**, 47, 558.
[4] G. Kresse, J. Hafner, *Phys. Rev. B* **1994**, 49, 14251.
[5] J. P. Perdew, K. Burke, M. Ernzerhof, *Phys. Rev. Lett.* **1996**, 77, 3865.
[6] P. E. Blöchl, *Phys. Rev. B* **1994**, 50, 17953.
[7] S. Grimme, *J. Comput. Chem.* **2006**, 27, 1787.
[8] Henkelman, G., Uberuaga, B. P. & Jonsson, H. , J. Chem. Phys. 113, 9901–9904 (2000).
